# TWEAK Signaling‐Induced ID1 Expression Drives Malignant Transformation of Hepatic Progenitor Cells During Hepatocarcinogenesis

**DOI:** 10.1002/advs.202300350

**Published:** 2023-04-21

**Authors:** Wenting Liu, Lu Gao, Xiaojuan Hou, Shiyao Feng, Haixin Yan, Hongyu Pan, Shichao Zhang, Xue Yang, Jinghua Jiang, Fei Ye, Qiudong Zhao, Lixin Wei, Zhipeng Han

**Affiliations:** ^1^ Tumor Immunology and Gene Therapy Center Third Affiliated Hospital of Naval Medical University Shanghai 200438 P. R. China; ^2^ Key Laboratory on Signaling Regulation and Targeting Therapy of Liver Cancer of Ministry of Education Eastern Hepatobiliary Surgery Hospital/National Center for Liver Cancer Naval Medical University Shanghai 200438 P. R. China; ^3^ Department of Urology Second Affiliated Hospital Anhui Medical University Hefei 230601 P. R. China; ^4^ Department of Hepatic Surgery Third Affiliated Hospital of Naval Medical University Shanghai 200438 P. R. China

**Keywords:** hepatic progenitor cells, inflammatory microenvironment, inhibitor of differentiation‐1, single‐cell RNA sequencing, tumor necrosis factor‐like weak inducer of apoptosis (TWEAK)

## Abstract

The malignant transformation of hepatic progenitor cells (HPCs) in the inflammatory microenvironment is the root cause of hepatocarcinogenesis. However, the potential molecular mechanisms are still elusive. The HPCs subgroup is identified by single‐cell RNA (scRNA) sequencing and the phenotype of HPCs is investigated in the primary HCC model. Bulk RNA sequencing (RNA‐seq) and proteomic analyses are also performed on HPC‐derived organoids. It is found that tumors are formed from HPCs in peritumor tissue at the 16th week in a HCC model. Furthermore, it is confirmed that the macrophage‐derived TWEAK/Fn14 promoted the expression of inhibitor of differentiation‐1 (ID1) in HPCs via NF‐*κ*B signaling and a high level of ID1 induced aberrant differentiation of HPCs. Mechanistically, ID1 suppressed differentiation and promoted proliferation in HPCs through the inhibition of HNF4*α* and Rap1GAP transcriptions. Finally, scRNA sequencing of HCC patients and investigation of clinical specimens also verified that the expression of ID1 is correlated with aberrant differentiation of HPCs into cancer stem cells, patients with high levels of ID1 in HPCs showed a poorer prognosis. This study provides important intervention targets and a theoretical basis for the clinical diagnosis and treatment of HCC.

## Graphical abstract

The dynamic process of HPCs under the inflammatory microenvironment from activation to malignant transformation is observed during hepatocarcinogenesis. The authors prove that macrophage‐derived TWEAK promotes ID1 expression, which suppresses differentiation and promotes cell proliferation in HPCs through inhibition of HNF4*α* and Rap1GAP transcription. The results may be facilitated to understand the potential mechanism of hepatocarcinogenesis induced by the inflammatory microenvironment.

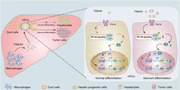

## 1 Introduction

Hepatocellular carcinoma (HCC) is one of the most common malignant tumors worldwide.^[^ ^1^ ^]^ It is estimated that 80% of all HCCs have a history of HBV and HCV infection.^[^ ^2^ ^]^ In addition, chronic inflammatory damage caused by alcoholic liver disease and non‐alcoholic fatty liver is also an important cause of liver cancer.^[^ ^3^ ^]^ Abundant evidence has confirmed that aberrant differentiation of hepatic progenitor cells (HPCs) in the inflammatory microenvironment is the origin of HCC.^[^ ^4^ ^]^ However, the dynamic changes and the potential molecular mechanisms underlying the malignant transformation of HPCs during the development of liver cancer are still elusive.

HPCs are referred to as oval cells in rodents, have bidirectional differentiation potential toward either hepatocytes or a biliary phenotype. HPCs normally reside in biliary ducts and can be activated by impairment of hepatocyte replicative potential during chronic liver damage.^[^ ^5^ ^]^ Thorgeirsson et al found that a distinct subtype of aggressive HCC expresses HPC markers, suggesting that this subtype of HCC might arise from HPCs.^[^ ^6^ ^]^ Markers of cancer stem cells (including EpCAM, CD133, CD24, and CD44), specific cytokeratins (including CK7 and CK19), CLDN4, and the transcription factor Sox9^[^ ^7^ ^]^ can be used to identify HPCs. It has been reported that HPCs are commonly accompanied by immune cells and cytokines in rodents.^[^ ^8^ ^]^ Our previous study found that the activation and aberrant differentiation of HPCs in the inflammatory microenvironment are the root causes of the occurrence and recurrence of liver cancer.^[^ ^4b^ ^]^

Here, we identified the HPCs subgroup by single‐cell RNA (scRNA) sequencing and investigated the phenotype of rat HPCs at different time points in the DEN‐induced primary HCC model. For further verification, bulk RNA sequencing (RNA‐seq) and proteomic analyses were also performed on organoids derived from primary HPCs. We first characterized the transcriptomic and proteomic profiles in HPCs at different time points in the primary HCC model. Further, we employed cell interaction analysis between non‐parenchymal cells and HPCs in the inflammatory microenvironment. We identified the molecular mechanism that drives the malignant transformation of HPCs. Taken together, our data promote an understanding of how the liver inflammatory microenvironment affects the function of HPCs and the malignant transformation of HPCs in hepatocarcinogenesis.

## 2 Results

### 2.1 Identification of HPCs by Single‐Cell RNA Sequencing during Hepatocarcinogenesis

**Figure 1:**
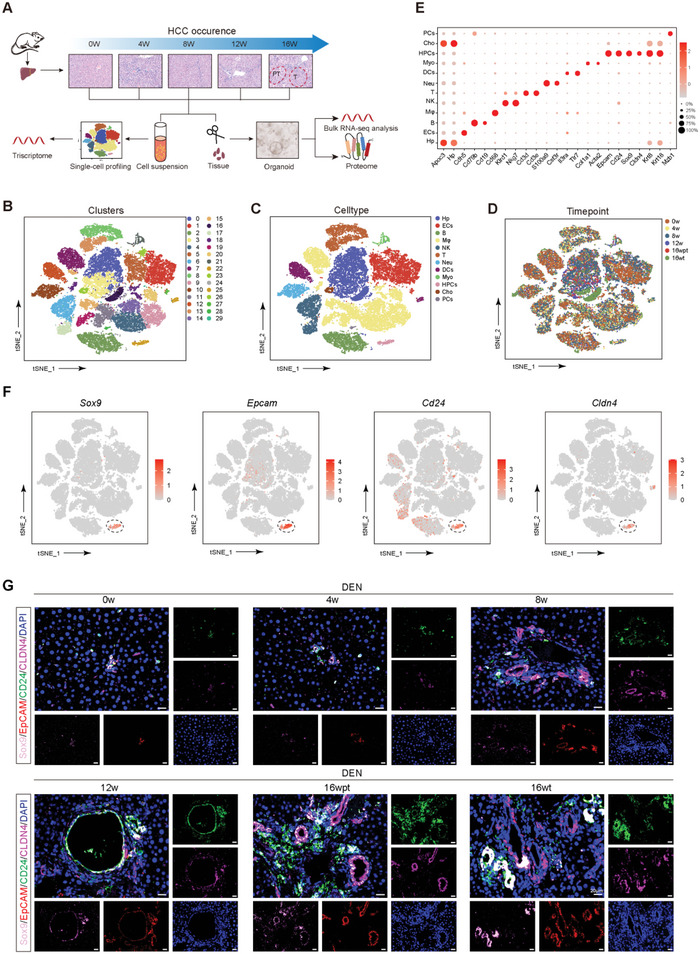
Identification of HPCs by single‐cell RNA sequencing during hepatocarcinogenesis. A) Schematic representation of the experimental strategy. At 16 weeks, two samples were collected: tumor tissue (16 wt) and adjacent non‐tumor tissue (16 wpt). B) T‐distributed stochastic neighbor embedding (t‐SNE) plot showing the clustering results for single cells from DEN‐treated rat liver. C) tSNE plot showing the cell types in the DEN‐treated rat liver, Hp: hepatocytes, ECs: endothelial cells, B: B cells, M*φ*: macrophage, NK: NK cells, T: T cells, Neu: neutrophils, DCs: dendritic cells, Myo: myofibroblasts, HPCs: hepatic progenitor cells, Cho: cholangiocytes, PCs: plasma cells. The colors represent the major cell types. D) tSNE plot showing single cells profiled in the present work colored by time points of DEN treatment. E) Dot plot showing the expression of marker genes in the indicated cell types. The dot size represents the percentage of cells expressing the marker genes in each major cell type, and the dot color represents the average expression level of the marker genes in each cell type. Red indicates high expression, while grey indicates low expression. F) t‐SNE plot showing the subgroup of HPCs expressing the marker genes *Sox9*, *Epcam*, *Cd24*, and *Cldn4*. G) Sox9 (pink), EpCAM (red), CD24 (green), and CLDN4 (hermosa rose) were detected in HPCs in rat liver at different time points of DEN treatment by immunofluorescence analysis. Nuclei were stained with DAPI (blue).

In order to analyze the potential mechanism underlying the malignant transformation of HPCs during hepatocarcinogenesis, we collected rat liver tissues at different time points (0, 4, 8, 12, and 16 weeks) of the primary HCC model for scRNA sequencing (one rat for each time point). Transcriptomic (two rats for each time point) and proteomic (one rat for each time point) analyses were also performed in primary HPC‐derived organoids for further verification (**Figure** **1**A). After quality control and removal of the batch effect (Figure S1A,B, Supporting Information), 37 930 single cells were clustered into 30 clusters. Clustering analysis revealed several major cell types in the liver (Figure 1B,C). All these cell subtypes were found in all the samples, albeit in different proportions (Figure 1D,E and Figure S1C, Supporting Information). Sox9^+^Epcam^+^Cd24^+^Cldn4^+^ HPCs were chosen for further analysis (Figure 1F and Figure S1D, Supporting Information). Compared to 0, 4, and 8 weeks, the infiltration levels of HPCs were greatly increased at 12 and 16 weeks of DEN treatment (Figure S1E, Supporting Information). Sox9^+^Epcam^+^Cd24^+^Cldn4^+^ HPCs were also observed in rat liver tissues during hepatocarcinogenesis, and the number of HPCs was also presented at an increased level at the 12th and 16th weeks of DEN treatment (Figure 1G).

### 2.2 Tumorigenicity of HPCs at Different Time Points during Hepatocarcinogenesis

**Figure 2:**
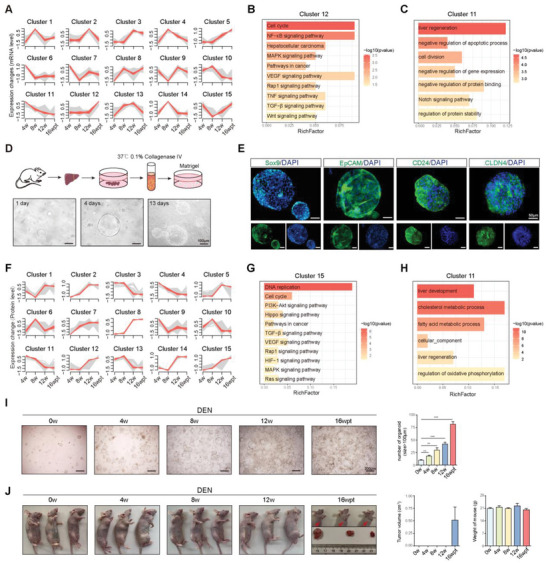
The tumorigenicity of HPCs at different time points during hepatocarcinogenesis. A) Fuzzy C‐means clustering identifies 15 distinct temporal patterns of gene expression. The *x*‐axis shows four developmental stages (4, 8, and 12 weeks, and 16 wpt), while the *y*‐axis shows the log2‐transformed, normalized intensity ratios at each stage. B,C) Bar graphs showing the pathways identified after KEGG and GO enrichment analysis of the genes in clusters 12 and 11, respectively. D) Primary HPCs were then isolated and cultured to form organoids. Pictures of HPC‐derived organoids were obtained at 1, 4, and 13 days. E) Expression of HPCs markers (Sox9, EpCAM, CD24, and CLDN4) was detected in HPC‐derived organoids by immunofluorescence analysis. Nuclei were stained with DAPI (blue). F) Fuzzy C‐means clustering identifies 15 distinct temporal patterns of protein expression. The *x*‐axis shows four developmental stages (4, 8, and 12 weeks, and 16 wpt), while the *y*‐axis shows the log2‐transformed, normalized intensity ratios at each stage. G,H) Bar graphs showing the pathways identified after KEGG and GO enrichment analysis of the proteins in clusters 15 and 11, respectively. I) Primary HPCs were obtained from rat livers at different time points of DEN treatment. The formation of HPC‐derived organoids was observed by microscopy and the number of organoids was calculated. Data presented as mean ± SD, **p < 0.01, ***p < 0.001. J) Tumorigenic potential of HPC‐derived organoids was observed. 200 HPC‐derived organoids were injected subcutaneously into the right axilla of the mice. Tumor volume and weight of mice were calculated and the data were presented as mean ± SD.

After we successfully used single‐cell sequencing technology to capture HPCs in the rat HCC model, we then observed the gene expression changes in HPCs at different time points during hepatocarcinogenesis. We applied the fuzzy C‐means algorithm to cluster the transcript expression profiles across all the developmental stages.^[^ ^9^ ^]^ In total, we observed 15 distinct clusters of temporal expression patterns (**Figure** **2**A and Table S1, Supporting Information). Among them, clusters 5 and 12 contain genes that are upregulated and cluster 11 contains downregulated genes, KEGG enrichment analysis was then performed (Figure 2A). The genes in clusters 5 and 12 were enriched in functions related to the following KEGG pathways: cell cycle, NF‐*κ*B, hepatocellular carcinoma, MAPK, pathways in cancer, VEGF, Rap1, TNF, TGF‐*β*, Wnt, AMPK, mTOR, PI3K‐Akt, HIF, and Hippo signaling (Figure 2B and Figure S2A, Supporting Information). Genes of cluster 11 were enriched in Gene ontology (GO) function: liver regeneration, negative regulation of apoptosis, cell division, and Notch signaling (Figure 2C). For further verifying the expression changes of the above‐mentioned signaling pathways at the mRNA and protein level, we established an organoid culture system for HPCs from rat liver (Figure 2D). As shown in Figure 2E, the HPC‐derived organoids stained positively for HPC markers (Sox9, EpCAM, CD24, and CLDN4). We also examined the differentiation potential of HPCs toward hepatocytes. The results demonstrated that HPC‐derived organoids were able to differentiate into hepatocytes with the expression of hepatocyte markers (Alb, Cyp2b1, Hnf3*β*, Hnf6, and Hnf4*α*) (Figure S2B,C, Supporting Information). Then we isolated primary HPCs at different time points of DEN treatment and total mRNA and protein were extracted for transcriptomic and quantitative proteomic analyses. At the mRNA level, fuzzy C‐means clustering identified eight distinct temporal patterns of gene expression. Cluster 7 contains genes showing upregulation and cluster 6 is downregulation (Figure S2D and Table S2, Supporting Information). KEGG enrichment analysis indicated that the activated pathways included HIF, pathways in cancer, TNF, PI3K‐AKT, mTOR, TGF‐*β*, cell cycle, and MAPK signaling (Figure S2E, Supporting Information). The downregulated functions were cell differentiation, liver regeneration, and Notch signaling (Figure S2F, Supporting Information). These are mostly consistent with the changes observed in the scRNA sequencing data. For proteomic analysis, there were also 15 distinct clusters of temporal protein expression patterns (Figure 2F and Table S3, Supporting Information). Upregulated proteins in clusters 12 and 15 and downregulated proteins in cluster 11 were selected for further KEGG and GO enrichment analysis. The activated signaling pathways were PI3K‐Akt, Hippo, pathway in cancer, TGF‐*β*, VEGF, Rap1, HIF, MAPK, Ras, and TNF signaling (Figure 2G and Figure S2G, Supporting Information). The inhibited function was liver development and regeneration (Figure 2H). Together, the transcriptome and proteome results suggest that the MAPK, Hippo, VEGF, PI3K‐Akt, HIF, TGF‐*β*, TNF, PI3K‐Akt, Rap1, and Ras signaling pathways may contribute to the regulation of HPC function and fate, the above signaling pathways were correlated with cell differentiation, proliferation, and response to the harsh hypoxic microenvironment. Additionally, the normal cell differentiation of HPCs was suppressed during HCC occurrence.

Then, the phenotype of HPCs at different time points was investigated. The formation of HPC‐derived organoids was observed by microscopy (Figure 2I). The tumorigenicity of HPCs was examined by assessing subcutaneous tumor formation in nude mice. We found that HPCs obtained from rat liver at 0, 4, 8, and 12 weeks did not form tumors. Tumors were formed from HPCs collected from the peritumor tissue of rats at the 16th week (16 wpt) of DEN treatment (Figure 2J). Then, we employed H&E and immunohistochemical (IHC) staining to identify the histological type of the HPCs‐derived tumors. The results were consistent with the HCC phenotype (Figure S2H, Supporting Information). These results indicate that hepatocarcinogenesis may originate from HPCs and the recurrence of HCC may also originate from HPCs in peritumor tissues.

### 2.3 Changes in the Transcriptomic and Proteomic Profile of Malignant HPCs

**Figure 3:**
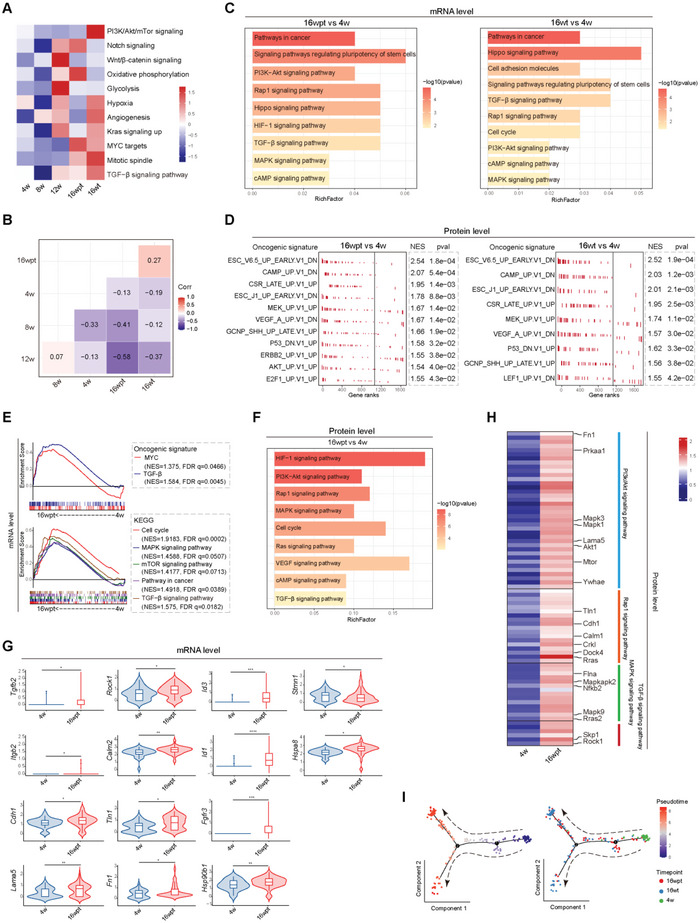
Changes in the transcriptomic and proteomic profile of malignant HPCs. A,B) Heatmap showing the enriched activated pathways and correlation in HPCs at different time points of DEN induction by GSVA analysis of scRNA seq data. C) Bar graphs showing the enriched activated pathways in HPCs from 16 wpt (left) and 16 wt (right) by scRNA sequencing (compared to 4 weeks). D) GSEA analysis showing proteomic sequencing‐based oncogenic signature in HPCs from 16 wpt (left) and 16 wt (right) by scRNA sequencing (compared to 4 weeks). E) Transcriptomic analysis was performed on organoids derived from HPCs from rats at 4w and 16 wpt. GSEA analysis of bulk mRNA was performed to detect the enriched pathway in HPCs from 16 wpt (compared to 4 weeks). F) Bar graph showing activated pathways in proteomic analysis of HPCs from 16 wpt (compared to 4w). G) Violin plots indicating expression of representative genes in the activated pathways in HPCs from 16 wpt (compared to 4w), *p < 0.05, **p < 0.01, ***p < 0.001, ****p < 0.0001. H) Heatmap showing expression levels of representative proteins in the activated pathways in HPCs from 16 wpt (compared to week 4). I) Trajectory of differentiation from HPCs (4w) into HPCs (16 wpt and 16 wt) predicted by monocle 2.

As shown in Figure 2J, HPCs collected from 16 wpt demonstrated tumor formation potential. Therefore, we further explore the potential signaling pathways that contribute to the malignant transformation of HPCs. GSVA analysis of scRNA sequencing data showed that oncogenic signaling Kras, Myc, and TGF‐*β* was upregulated in HPCs from 16 wpt and tumor tissue of rats treated with DEN for 16 weeks (16 wt) (**Figure** **3**A). And expression profile in HPCs from 16 wpt was most correlated with that from 16 wt, which suggested that HPCs from 16 wpt presented the expression features of cancer stem cells (CSCs) (Figure 3B).

In order to investigate the change of expression profile in HPCs during hepatocarcinogenesis, GO and KEGG enrichment analyses were performed on the differentially expressed genes (DEG) and proteins between different time points. Compared to HPCs collected at the 4th week, GO analysis revealed that the upregulated genes at the 8th week were mainly enriched in functions related to positive regulation of cell adhesion, positive regulation of cell migration, and cell division (Figure S3A, Supporting Information, left). KEGG enrichment analysis showed that the genes were enriched in pathways related to focal adhesion, collagen formation, and G2/M checkpoints (Figure S3A, Supporting Information, right). At the protein level, focal adhesion, liver regeneration, cell differentiation, and cell migration were also enriched (Figure S3B,C, Supporting Information). HPCs are mainly responsible for repairing liver injury, which indicates that they respond to DEN‐induced liver damage by increasing their proliferation and differentiation potential. At 12 weeks, DEG enrichment analysis found that many pathways were activated, including Hippo, PI3K‐AKT, TGF‐*β*, HIF‐1, PPAR, VEGF, PDGF, FGFR2, Hedgehog signaling, and cell cycle (Figure S3D, Supporting Information). At the protein level, enrichment analysis identified that HIF, PI3K‐AKT, mTOR, Rap1, VEGF, MAPK, Hippo, and Hedgehog signaling pathways were activated (Figure S3E, Supporting Information). The results suggested that the proliferation and differentiation of HPCs were further activated, and the HPCs showed a phenotype consistent with response to the harsh hypoxic microenvironment. However, tumors were not visible in rat livers after 12 weeks. Therefore, HPCs do not possess a tumorigenic phenotype in vivo, while the genotype of HPCs is already in a state of instability.

When the DEN treatment time was extended to 16 weeks, visible tumors were formed in the rat livers. Gene expression profile analysis was performed on HPCs from 16 wpt. DEG enrichment analysis revealed that pathways in cancer, PI3K‐AKT, Rap1, Hippo, TGF‐*β*, MAPK, and cAMP signaling were activated, which was consistent with the HPCs from 16 wt (Figure 3C). At the protein level, GSEA analysis of the oncogenic signatures from the Molecular Signatures Database (MSigDB) further validated our gene expression‐based index and confirmed HPCs from 16 wpt presented oncogenic differentiation (Figure 3D). Expression of CSCs’ markers was also upregulated in HPCs from 16 wpt (Figure S3F, Supporting Information). The results indicate a tumor‐initiating phenotype in HPCs from 16 wpt. GSEA analysis of bulk RNA sequencing data also demonstrated the activation of Myc and TGF‐*β*, activated pathways included cell cycles, MAPK, mTOR, and pathways in cancer were also verified in HPCs from 16 wpt by GSEA analysis (Figure 3E). Further proteomics analysis indicated that the HIF‐1, PI3K‐AKT, Rap1, MAPK, Ras, VEGF, cAMP, and TGF‐*β* signaling pathways were significantly upregulated in HPCs from 16 wpt (Figure 3F). TGF‐*β*, Rap1, PI3K‐AKT, and MAPK signaling were activated at both the mRNA and protein levels (Figure 3G,H). The above data suggested that TGF‐*β*, Rap1, PI3K‐AKT, and MAPK signaling significantly may contribute to the malignant transformation state of HPCs. To explore the relationship among HPCs from week 4, 16 wpt, and 16 wt, we constructed a transcriptional trajectory of these cells on a pseudotime scale using a monocle.^[^ ^10^ ^]^ HPCs from week 4 (normal) were distributed at one end of the pseudo‐temporal trajectory whereas HPCs from 16 wpt (malignant state) and 16 wt (CSCs) resided at the other end, suggesting that CSCs might derive from normal HPCs (Figure 3I).

### 2.4 ID1 Is Predicted to Drive the Malignant Transformation of HPCs

**Figure 4:**
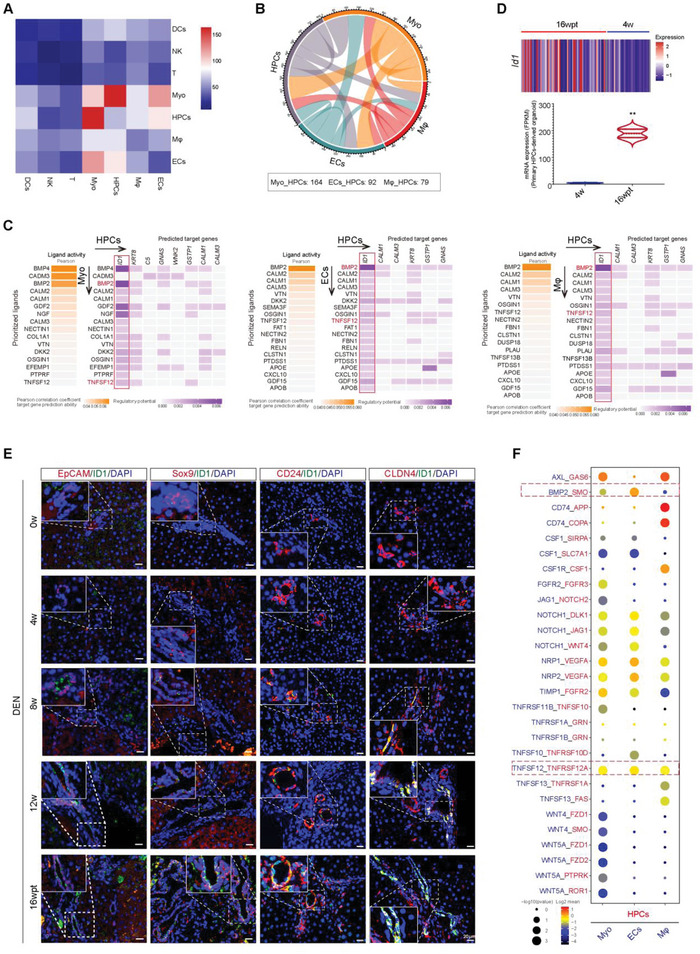
ID1 is predicted to drive the malignant transformation of HPCs. A) Heatmap showing the number of ligands and receptors involved in significant L‐R pairs between different cell types in 16 wpt by CellphoneDB. B) Chord diagrams of cellular interactome between endothelial cells, myofibroblasts, and macrophage from 16 wpt. C) NicheNet interaction heatmap between HPCs and myofibroblasts, endothelial cells and macrophage. Note the BMP2 and TNFSF12‐specific induction of ID1 in HPCs from 16 wpt. D) Heatmap showing mRNA level of *Id1* in HPCs at 4 w and 16 wpt by scRNA sequencing. Violin plots indicating *Id1* expression in primary HPC‐derived organoids at 4 w and 16 wpt by bulk RNA‐seq analysis, **p < 0.01. E) ID1 expression (green) was detected in HPCs (red) at different time points of DEN induction by immunofluorescence analysis. Nuclei were stained with DAPI (blue). F) Dot plot of interactions between HPCs and myofibroblasts, endothelial cells and macrophages from 16 wpt according to CellPhoneDB analysis. Color indicates the scaled mean expression level of ligand and receptor (Mol1/2). Blue indicates low‐intensity interaction and red indicates high‐intensity interaction. The dot size represents −log10 (*p*‐value), and a larger point indicates a smaller *p‐*value.

Several studies have confirmed that the differentiation and function of HPCs are regulated by the liver microenvironment.^[^ ^11^ ^]^ To investigate the interactions between HPCs and liver microenvironment in HCC occurrence, we utilized a set of ligand–receptor (L‐R) pairs to gain insights into the regulatory relationships among HPCs, myofibroblasts, endothelial cells, and immune cells. Based on this analysis, myofibroblasts, endothelial cells, and macrophages showed strong potential interactions with HPCs of 16 wpt (**Figure** **4**A,B). In order to further explore the potential mechanism underlying the malignant differentiation of HPCs, we performed NicheNet analysis, which allowed us to predict cellular interactions by linking ligands and target gene expression in HPCs of 16 wpt.^[^ ^12^ ^]^ Interestingly, the results showed that inhibitor of differentiation‐1 (ID1) in HPCs can be activated by ligands (BMP2 and TNFSF12) derived from cells in the liver microenvironment, including myofibroblasts, endothelial cells, and macrophage (Figure 4C). Id1 is a stem cell‐like gene and is overexpressed in several types of cancers. Compared to HPCs from 4w, *Id1* expression was significantly increased in HPCs from 16 wpt at both scRNA sequencing data and mRNA level from primary HPCs (Figure 4D). We then validated these observations by fluorescence co‐staining experiments and observed increased expression of ID1 in HPCs derived from 16 wpt compared to normal liver and liver at 4, 8, and 12 weeks (Figure 4E). In order to further decipher the receptor‐ligand interactions in myofibroblasts, endothelial cells, macrophages, and HPCs, we performed cell–cell interaction analysis; the data showed that TNFSF12 and BMP2 might regulate ID1 expression through binding to related receptors including TNFRSF12A and SMO in HPCs (Figure 4F). These results strongly imply that ID1 may play a key role in the malignant transformation of HPCs induced by microenvironment‐associated cells.

### 2.5 Macrophage‐Derived TWEAK Promoted ID1 Expression through Activation of NF‐*κ*B Signaling

**Figure 5:**
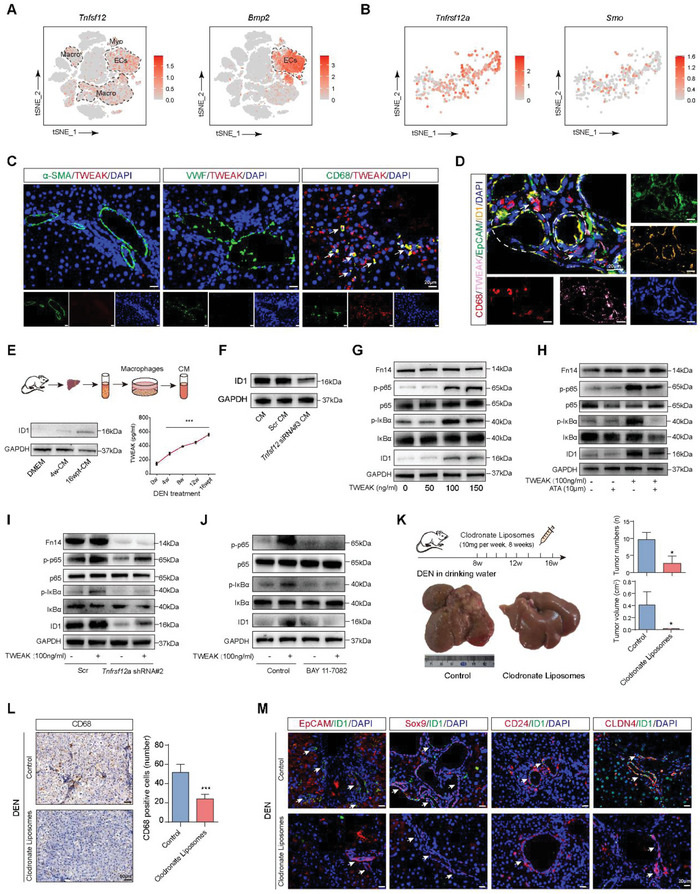
Macrophage‐derived TWEAK promoted ID1 expression through activation of NF‐*κ*B signaling. A) tSNE plot showing the expression of *Tnfsf12* and *Bmp2* in myofibroblasts, endothelial cells, and macrophages. B) tSNE plot showing the expression of *Tnfrsf12a* and *Smo* in HPCs. C) TWEAK expression (red) was detected in myofibroblasts (*α*‐SMA), endothelial cells (VWF), and macrophages (CD68) from 16 wpt by immunofluorescence analysis. Nuclei were stained with DAPI (blue). D) The co‐location of TWEAK‐expressing (pink) in macrophages (CD68, red) with ID1‐expressing (yellow) HPCs (EpCAM, green) was detected by immunofluorescence analysis. Nuclei were stained with DAPI (blue). E) Macrophages derived from rats with different time points of DEN treatment were collected and conditioned medium (CM) from week 4 and 16 wpt was used to treat HPCs. ID1 expression was examined by western blotting (lower left panel). TWEAK level was measured in macrophages derived from different time points of DEN treatment by ELISA kit (lower right panel), data are presented as mean ± SD. ***p < 0.001. F) CM derived from *Tnfsf12*‐siRNA‐treated‐macrophages from 16 wpt was collected and then used to treat HPCs. ID1 expression was examined by western blotting. GAPDH was used as the internal reference. G) HPCs were treated with different concentrations of TWEAK (0, 50, 100, and 150 ng mL^−1^) respectively and then the expression of Fn14, p‐p65, p65, p‐I*κ*B*α*, I*κ*B*α*, and ID1 was detected by western blotting in each group. GAPDH was used as the internal reference. H) TWEAK (100 ng mL^−1^) and ATA (10 µm) were used to treat HPCs and then the expression of Fn14, p‐p65, p65, p‐I*κ*B*α*, I*κ*B*α*, and ID1 was detected by western blotting in each group. GAPDH was used as the internal reference. I) TWEAK was used to treat the HPCs that the expression of Fn14 was inhibited by Tnfrsf12a‐shRNA#2 and then the expression of Fn14, p‐p65, p65, p‐I*κ*B*α*, I*κ*B*α*, and ID1 was detected by western blotting in each group. GAPDH was used as the internal reference. J) TWEAK (100 ng mL^−1^) was used to treat HPCs with or without BAY 11–7082 and then the expression of p‐p65, p65, p‐I*κ*B*α*, I*κ*B*α*, and ID1 was detected by western blotting in each group. GAPDH was used as the internal reference. K) Rats of DEN‐treated 8 weeks received a single injection of clodronate liposome into the tail vein per week to eliminate macrophages, HCC occurrence was observed in different groups, and tumor number and volume were calculated in each group, *p < 0.05. L) CD68 expression was detected by IHC in different groups. The number of positive cells was calculated in each group, data are presented as mean ± SD. ***p < 0.001. M) ID1 expression (green) was detected in HPCs (red) in different groups by immunofluorescence analysis. Nuclei were stained with DAPI (blue). The white arrow indicates the positive location.

To further investigate the mechanism involved in microenvironment‐associated cells‐induced ID1 expression, we observed the expression of *Tnfsf12* and *Bmp2* in myofibroblasts, endothelial cells, and macrophages, which induced the upregulation of ID1 in HPCs from 16wpt. As shown in **Figure** **5**A, *Tnfsf12* was upregulated in myofibroblasts, endothelial cells, and macrophages, and *Bmp2* was mainly upregulated in myofibroblasts and endothelial cells. The receptor of *Tnfsf12* (*Tnfrsf12a*) is highly expressed in HPCs, however, the expression of *Smo* was at a low level (Figure 5B), which suggested that *Tnfsf12* mainly contributed a role in HPCs. The Tnfsf12 gene is primarily responsible for encoding tumor necrosis factor‐like weak inducer of apoptosis (TWEAK) in cells, which is a secretory protein. Next, we detected the expression of TWEAK in myofibroblasts, endothelial cells, and macrophages, and found that macrophages mainly produced TWEAK in 16wpt (Figure 5C). The co‐location of TWEAK‐expressing macrophages with ID1‐expressing HPCs was also confirmed by fluorescence co‐staining analysis (Figure 5D). Then, we isolated rat liver macrophages at different time points of DEN treatment and a conditioned medium (CM) of macrophages from 16 wpt was collected, we observed that the CM could effectively upregulate ID1 expression in HPCs (Figure 5E). It was found that the level of TWEAK secreted by macrophages from 16 wpt was significantly increased by ELISA examination (Figure 5E), suggesting that TWEAK was mainly derived from macrophages in the liver microenvironment. To further demonstrate the role of TWEAK in regulating ID1 expression in HPCs, we used *Tnfsf12* siRNA to block TWEAK secretion in macrophages (Figure S3G, Supporting Information), and ID1 expression in HPCs was reduced when treated with CM of *Tnfsf12* siRNA‐disposed macrophages from 16 wpt (Figure 5F). HPCs from 8 weeks was isolated and pre‐treated with TWEAK, HPCs that received 100 and 150 ng mL^−1^ TWEAK for 24 h exhibited significantly increased level of phosphorylated p65, I*κ*B*α*, and ID1 (Figure 5G). Next, aurintricarboxylic acid (ATA), an inhibitor of TWEAK/fibroblast growth factor‐inducible 14 (Fn14)/NF‐*κ*B signaling,^[^ ^13^ ^]^ was used to determine whether Fn14‐NF‐*κ*B signaling was associated with the mechanism of TWEAK‐mediated ID1 upregulation. As presented in Figure 5H, the results demonstrated that TWEAK‐mediated ID1 upregulation is inhibited by ATA treatment (Figure 5H). To further confirm the role of TWEAK/Fn14/NF‐*κ*B signaling in the upregulation of ID1 expression, *Tnfrsf12a* shRNA and BAY 11–7078 (inhibitor of NF‐*κ*B signaling) were used to suppress TWEAK/Fn14/NF‐*κ*B signaling in HPCs, which lead to the non‐effectiveness of ID1 expression when HPCs treated with TWEAK (Figure 5I,J and Figure S3H, Supporting Information), suggesting that TWEAK/Fn14/NF‐*κ*B signaling serves a key role in ID1 expression in HPCs.

In order to further verify the role of macrophage‐derived TWEAK‐induced ID1 up‐regulation in HCC occurrence, rats DEN‐treated for 8 weeks received a single injection of clodronate liposome from the tail vein per week to eliminate macrophages (Figure 5K,L). HCC occurrence was significantly inhibited after treatment with clodronate liposome (Figure 5K), and further investigation found that treatment of clodronate liposome significantly decreased the expression of ID1 in HPCs (Figure 5M). The results indicate that macrophage‐derived TWEAK promotes ID1 expression, which mediates the malignant transformation of HPCs through the Fn14/NF‐*κ*B pathway.

### 2.6 ID1 Suppresses Differentiation and Promotes Cell Proliferation in HPCs through Inhibition of HNF4*α* and Rap1GAP Transcription

**Figure 6:**
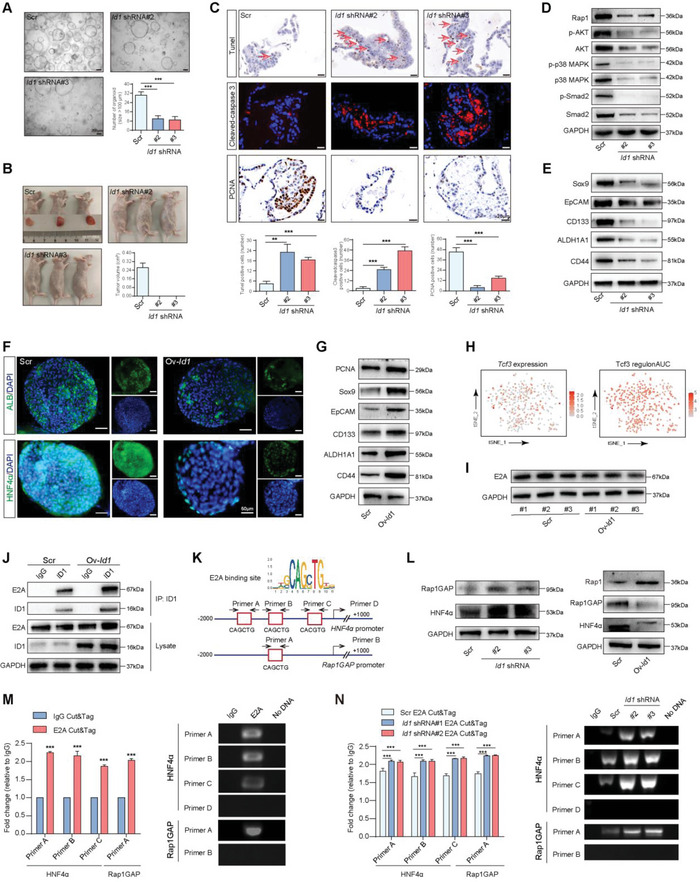
ID1 suppresses differentiation and promotes cell proliferation in HPCs through the inhibition of HNF4*α* and Rap1GAP transcription. A) Rat liver HPCs were treated with lentivirus expressing scramble shRNA (Scr), *Id1* shRNA#2, or *Id1* shRNA#3. HPC‐derived organoid formation in each group was observed by microscopy. The number of organoids was calculated in each group, data are presented as mean ± SD. ***p < 0.001. B) Tumorigenic potential of HPC‐derived organoids in each group was assessed by subcutaneous injection of organoids into the right axilla of mice. C) Top: TUNEL staining was performed to investigate the apoptosis of HPCs in each group. Middle and bottom: cleaved caspase3 and PCNA were detected by immunofluorescence and IHC analysis, respectively, in each group. The number of positive cells was calculated in each group, data are presented as mean ± SD. **p < 0.01, ***p < 0.001. The red arrow indicates the positive location. D) The expression of ID1 in HPC‐derived organoids was inhibited by shRNA and the levels of cell proliferation‐related signal pathways were examined in each group by western blotting. GAPDH was used as the internal reference. E) The expression of ID1 in HPC‐derived organoids was inhibited by shRNA and the expression of stem cell markers was evaluated in each group by western blotting. GAPDH was used as the internal reference. F) The in vitro differentiation induction system was performed in HPC‐derived organoids in each group. Protein levels of ALB and HNF4*α* (green) were examined by immunofluorescence analysis. Nuclei were stained with DAPI (blue). G) ID1 was overexpressed in HPC‐derived organoids and the expression of PCNA and stem cell markers was evaluated in each group by western blotting. GAPDH was used as the internal reference. H) t‐SNE plot showing the expression and regulatory activity of TCF3 in HPCs from the liver of rat HCC model by scRNA sequencing. I) HPC‐derived organoids were isolated and cultured from three rats respectively and the ID1 was overexpressed in these HPC‐derived organoids. Expression levels of E2A were evaluated in each group by western blotting. GAPDH was used as the internal reference. (#1, #2, and #3 represent three different HPC‐derived organoids samples) J) The binding of ID1 and E2A was detected by IP assay. GAPDH was used as the internal reference. K) Top: the consensus binding site of E2A. Bottom: E2A binding sites in the *HNF4α* and *Rap1GAP* promoter, predicted by JASPAR. L) The expression of ID1 in HPC‐derived organoids was up or down‐regulated respectively and Rap1, Rap1GAP, and HNF4*α* expression was examined in each group by western blotting. GAPDH was used as the internal reference. M) Enrichment of the fragments containing the E2A binding sites within the *HNF4α* and *Rap1GAP* promoter in HPCs by CUT&Tag‐qPCR (right) and agarose gel electrophoresis analysis (left). Fold enrichment is relative to the background DNA fragment pulled down by IgG immunoprecipitation. Data are presented as mean ± SD. ***p < 0.001. N) Enrichment of the fragments containing the E2A binding sites within the *HNF4α* and *Rap1GAP* promoter in HPCs from different groups by CUT&Tag‐qPCR (right) and agarose gel electrophoresis analysis (left). Fold enrichment is relative to the background DNA fragment pulled down by IgG immunoprecipitation. Data are presented as mean ± SD. ***p < 0.001.

For investigating the mechanism involved in ID1‐induced malignant transformation of HPCs, HPCs were isolated from 16 wpt, cultured in organoid form in vitro, and transfected with *Id1* shRNA lentivirus (Figure S4A, Supporting Information). The results showed that *Id1* shRNA effectively reduced the expression of ID1 in HPCs (Figure S4B, Supporting Information). Knocking down the expression of ID1 in HPCs greatly inhibited the formation of organoids derived from HPCs (**Figure** **6**A). We further observed the effect of inhibiting the expression of ID1 on the tumorigenicity of HPCs. As shown in Figure 6B, the tumorigenic potential of HPCs was suppressed by knocking down the expression of ID1. TUNEL staining revealed increased apoptosis of HPCs in the ID1‐knockdown group. Immunofluorescence and IHC data indicated upregulation of cleaved caspase3 and downregulation of PCNA (Figure 6C). Immunoblot analysis showed marked down‐regulation of proteins in cell proliferation‐related signaling pathways, including the Rap1, PI3K‐AKT, MAPK, and TGF‐*β* signaling pathways which were activated in malignant HPCs, in HPCs with ID1 knockdown (Figure 6D and Figure S4C, Supporting Information). Besides that, we also observed the effect of TWEAK on the expression of Rap1, PI3K‐AKT, MAPK, and TGF‐*β* signaling pathways. We found that TWEAK could effectively lead to the activation of Rap1, PI3K‐AKT, MAPK, and TGF‐*β* signaling pathways (Figure S4D, Supporting Information). These results strongly imply that TWEAK‐induced upregulation of ID1 may lead to the malignant transformation of HPCs by promoting cell proliferation and suppressing cell apoptosis. ID1 was first reported to be expressed in stem cells and to inhibit the maturation of stem cells.^[^ ^14^ ^]^ Therefore, we investigated the effect of ID1 on the stemness of HPCs. As shown in Figure 6E, the expression of stem cell markers, including Sox9, EpCAM, CD133, ALDH1A1, and CD44, was greatly reduced in HPCs with ID1 shRNA transfection compared to the scramble group.

In order to further validate the influence of ID1 on the differentiation of HPCs, we designed a lentivirus to overexpress ID1 and transfected it into HPCs from 8w (Figure S4E,F, Supporting Information). Further in vitro differentiation experiments confirmed that overexpression of ID1 notably suppressed the expression of the hepatocyte markers Alb, Cyp2b1, Hnf3*β*, Hnf6, and Hnf4*α* (Figure S4G, Supporting Information and Figure 6F). This suggests that ID1 inhibits the differentiation of HPCs into hepatocytes. Compared with the scramble group, overexpression of ID1 promoted the expression of PCNA and stem cell markers (Figure 6G). Taken together, our results reveal that ID1 inhibits the differentiation and enhances the stemness of HPCs, thereby promoting the malignant transformation of HPCs by increasing cell proliferation while decreasing cell apoptosis.

Id1 proteins lack a DNA binding domain, and they function as dominant negative regulators of basic HLH transcriptional factors through heterodimerizing with other bHLH factors such as E2A and inhibiting their binding to DNA.^[^ ^14^ ^]^ We performed SCENIC analysis for the investigation of transcriptional factors in HPCs from scRNA sequencing data.^[^ ^15^ ^]^ The results indicated that E2A transcriptional factors family members (*TCF3* and *TCF12*) were expressed in HPCs from rat model, SCENIC also identified a network of TCF3 and TCF12 in HPCs (Figure 6H and Figure S4H, Supporting Information). The in vitro investigation also demonstrated the positively expressed protein E2A was presented in HPCs (Figure 6I). IP assay indicated that ID1 could bind to protein E2A (Figure 6J). The JASPAR website predicted that the E2A motif can bind to three binding sites in the hepatocyte nuclear factor 4*α* (*HNF4α*) promoter and one binding site in the Rap1 GTPase‐activating protein (*Rap1GAP*) promoter (Figure 6K). HNF4*α* is a nuclear receptor that plays an important role in mediating the differentiation of HPCs. Rap1GAP has been reported to as a negative regulator of Rap1 activity and serves an important role in tumor cell proliferation.^[^ ^16^ ^]^ The results demonstrated that silencing Id1 expression could increase HNF‐4*α* and Rap1GAP protein levels, and overexpression of Id1 could suppress the level of HNF‐4*α* and Rap1GAP (Figure 6L). CUT&Tag assays revealed that E2A binds to three high‐affinity E‐boxes in the *HNF4α* promoter and one in the *Rap1GAP* promoter (Figure 6M). The binding of E2A to the promoters of *HNF4α* and *Rap1GAP* was enhanced after ID1 was knockdown (Figure 6N). The transcription of HNF4*α* and Rap1GAP was also suppressed when E2A was knockdown by shRNA (Figure S4I,J, Supporting Information), which indicates that ID1 inhibits *HNF4α* and *Rap1GAP* transcription through binding to protein E2A. Taken together, our results reveal that ID1 inhibits the differentiation of HPCs and promotes cell proliferation via suppression of *HNF4α* and *Rap1GAP* transcription, thereby promoting the malignant transformation of HPCs.

### 2.7 The Correlation between ID1 Expression in HPCs of Clinical Specimens and Prognosis of HCC Patients

**Figure 7:**
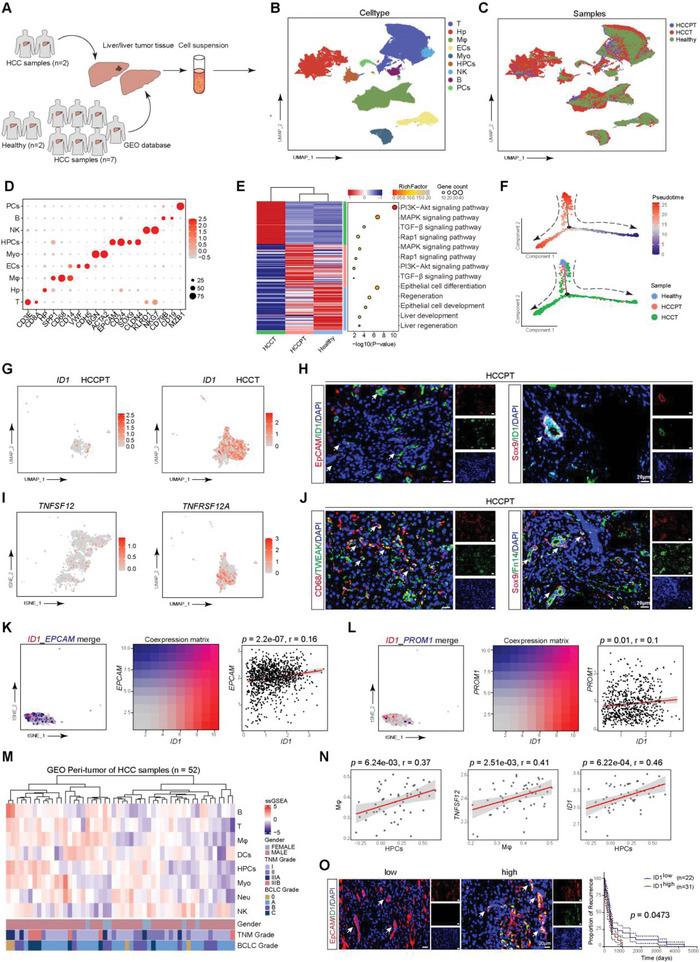
The correlation between ID1 expression in HPCs of clinical specimens and prognosis of HCC patients. A–C) t‐SNE plot showing the annotation and color codes for cell types and samples in the liver from a healthy donor (*n* = 2), HCC adjacent non‐tumor (HCCPT, *n* = 7), and tumor tissue (HCCT, *n* = 9). D) Dot plot showing the expression of marker genes in the indicated cell types. The dot size represents the percentage of cells expressing the marker genes in each major cell type, and the dot color represents the average expression level of the marker genes in each cell type. Red indicates high expression, while grey indicates low expression. E) Heatmap and dot plot showing the upregulated gene and enriched activated pathways in HPCs from the liver of healthy, HCCPT, and HCCT by scRNA sequencing. F) Trajectory of differentiation from HPCs (healthy) into HPCs (HCCPT and HCCT) predicted by monocle 2. G) UMAP plot indicating *Id1* expression in HPCs from the liver of HCCPT and HCCT samples. H) ID1 expression (green) in HPCs (EpCAM and Sox9, red) from the liver of HCCPT was detected by immunofluorescence analysis. Nuclei were stained with DAPI (blue). I) tSNE and UMAP plots showing the expression of TNFSF12 and TNFRSF12A in macrophage and HPCs from the liver of HCCPT samples. J) TWEAK and Fn14 expression (green) in macrophages (CD68, red) and HPCs (Sox9, red) from the liver of HCCPT was detected by immunofluorescence analysis. Nuclei were stained with DAPI (blue). K,L) tSNE plots showing the co‐expression of EPCAM, PROM1 (CD133), and ID1 in HPCs. Red/blue dots represent ID1+ only/EPCAM+ or PROM1+ only, respectively, and pink dots indicate HPCs co‐expressing ID1 and EPCAM or PROM1; the middle panel showing the co‐expression matrix across HPCs with different expressions of ID1 and EPCAM or PROM1; right panel showing the correlation (Pearson) between ID1 and EPCAM or PROM1 in HPCs. M) Heatmap of cell abundance predicted per sample from GEO peritumor tissues of HCC cohort by ssGSEA. Shown are row *z*‐score. N) Correlation between macrophages and HPCs, TNFSF12 level and macrophages, and ID1 level and HPCs in peri‐tumor tissues of HCC. Coefficient was calculated with spearman correlation analysis. O) ID1 expression (green) was detected in HPCs (red) in adjacent non‐tumor liver tissue of HCC patients by immunofluorescence analysis. Nuclei were stained with DAPI (blue). Recurrence rate was analyzed in each group of HCC patients (Kaplan–Meier method). The *p*‐value was obtained by the log‐rank multiple comparisons test.

We have demonstrated that ID1 plays a key role in the malignant transformation of HPCs during hepatocarcinogenesis in animal HCC models, and then we investigated the relationship between ID1 expression in HPCs and aberrant differentiation of HPCs as well as the prognosis of HCC patients. We performed scRNA sequencing of liver tumors from 2 HCC patients and downloaded liver scRNA sequencing data of healthy donor (*n* = 2), HCC adjacent tumor (*n* = 7), and tumor samples (*n* = 7) from GEO databases (**Figure** **7**A–C). HPCs subset was identified with *SOX9*, *EPCAM*, *CD24*, and *CLDN4* (Figure 7D and Figure S5A, Supporting Information). DEG enrichment analysis indicated that Rap1, PI3K‐AKT, MAPK, and TGF‐*β* signaling was also activated in HPCs from adjacent tumor and tumor tissue of clinical samples, epithelial cell differentiation, liver regeneration, and development was enriched in HPCs from healthy cases (Figure 7E), which suggested the signaling pathway mentioned above might contribute to the malignant transformation of HPCs into CSCs. Pseudotime analysis also suggested HPCs from HCCPT and HCCT were diverging from the HPCs of healthy samples (Figure 7F). ID1 expression was observed in HPCs from HCCPT and HCCT samples (Figure 7G,H), which was consistent with the results that were found in the rat HCC model. *TNFSF12* was mainly derived from macrophage and *TNFRSF12A* was also found in HPCs from HCCPT samples (Figure 7I,J, and Figure S5B, Supporting Information). Furthermore, co‐expression analysis demonstrated that ID1‐high HPCs are largely overlapping with EPCAM, PROM1, and CD44‐high HPCs, and there is a significant correlation between ID1 and EPCAM, PROM1, and CD44 in HPCs from HCCPT and HCCT samples (Figure 7K,L and Figure S5C, Supporting Information). We used the ssGSEA approach to deconvolve the relative abundance of each cell type based on expression profiling data retrieved from the GEO database^[^ ^17^ ^]^ (Figure 7M). Based on this analysis, we found a significant correlation between the level of macrophages and level of activated HPCs (*p* = 6.24e‐03, *r* = 0.37), TNFSF12 expression and level of macrophage (*p* = 2.51e‐03, *r* = 0.41), ID1 expression and the level of HPCs activation (*p* = 6.22e‐04, *r* = 0.46) in HCC adjacent non‐tumor tissues (Figure 7N). These results suggest that macrophage‐derived TWEAK may contribute to the ID1 expression and HPCs proliferation. We further examined the expression of ID1 in HPCs in adjacent non‐tumor tissues in clinical patients and divided the patients into high‐expression and low‐expression groups. Recurrence analysis revealed that the ID1 high expression group had a shorter recurrence time than the ID1 low expression group (Figure 7O), which suggested that ID1 expression was correlated with malignant transformation of HPCs and recurrence of HCC. We also verified the positive correlation between *ID1* expression and *SOX9* (*p* = 6.90e‐05, *r* = 0.20), *EPCAM* (*p* = 5.37e‐06, *r* = 0.23), *CD24* (*p* = 1.82e‐07, *r* = 0.27), and *CLDN4* (*p* = 6.04e‐05, *r* = 0.18) expression in HCC tumor tissue from TCGA datasets (Figure S5D, Supporting Information). The results of survival analysis showed that SOX9 and ID1 double positive samples demonstrated a poorer overall survival time compared with SOX9 and ID1 double negative group. Besides that, the combination of CLDN4 and ID1 also indicated a poorer OS in HCC patients (Figure S5E, Supporting Information). We performed drug sensitivity testing in liver tumor organoids derived from 5 HCC patients. There were three patients (HCC‐2, HCC‐3, and HCC‐5) showed resistance to sorafenib treatment and two samples (HCC‐1 and HCC‐4) were sensitive (Figure S5F, Supporting Information). IHC data showed that HCC‐2, HCC‐3, and HCC‐5 cases presented a high level of ID1 expression (Figure S5G, Supporting Information). These results strongly imply that ID1 was correlated with the prognosis of HCC patients.

## 3 Discussion

It has been confirmed that aberrant differentiation of HPCs in the inflammatory microenvironment is the origin of HCC. However, the dynamic changes and the potential molecular mechanisms underlying the malignant transformation of HPCs in the inflammatory microenvironment during the development of HCC are still elusive. Here, the DEN‐induced primary rat HCC model can well simulate chronic inflammatory damage‐induced liver fibrosis, cirrhosis, and ultimately liver cancer, which is consistent with the pathogenesis of clinical HCC. We generated a comprehensive single‐cell atlas of the liver to understand the cellular landscape from early development to terminal disease. The dynamic changes of the transcriptome and proteome in HPCs during the formation of HCC were further analyzed. We found that macrophage‐derived TWEAK‐promoted ID1 expression plays a key role in regulating the proliferation, differentiation, and malignant transformation of HPCs in hepatocarcinogenesis. The expression of ID1 in HPCs in clinical samples is negatively correlated with the recurrence and prognosis of HCC patients. Our study represents an essential step toward understanding how HPCs initiate tumor occurrence and reveals the existence of active crosstalk between HPCs and the inflammatory microenvironment in HCC.

As reservoir cells, HPCs were shown to be activated in a wide range of liver diseases.^[^ ^18^ ^]^ The presence of HPCs in primary liver cancers, therefore, raised the suspicion that they may be implicated in hepatocarcinogenesis. Related theories emerged that included maturation arrest and dedifferentiation as mechanisms. Here, we first described the dynamic changes of HPCs at different time points during the occurrence of liver cancer. We isolated primary HPCs from rats at the early, middle, and late stages of DEN‐induced primary hepatocarcinogenesis. Only HPCs collected from peritumor tissue at the late stage (16 weeks) caused a malignant phenotype. HPCs were activated in the early stage of HCC occurrence and participated in the repair of liver damage. The instability of HPCs in the process of proliferation and differentiation increased when chronic liver injury persisted. HPCs continued to proliferate while losing the ability to differentiate into hepatocytes, thus initiating tumorigenesis. scRNA sequencing, bulk RNA‐seq, and proteomic analyses in primary HPCs suggest that the TGF‐*β*, Rap1, PI3K‐AKT, and MAPK signaling pathways may make a major contribution to the malignant transformation of HPCs. TGF‐*β* signaling plays an important role in tumor initiation by controlling numerous cellular functions including proliferation, apoptosis, and differentiation.^[^ ^19^ ^]^ The Rap1 and PI3K‐AKT signaling pathways exert a wide range of biological effects in tumorigenesis, including anti‐apoptotic effects and the promotion of cell survival.^[^ ^20^ ^]^ Abnormal or excessive activation of the MAPK signaling pathway plays an important role in the malignant transformation and evolution of cells.^[^ ^21^ ^]^

The inflammatory microenvironment plays an important role in regulating the activation and function of HPCs during chronic liver injury. In the chronically injured rodent liver, oval cells are commonly accompanied by immune cells and cytokines.^[^ ^11c^ ^]^ Furthermore, the infiltration of inflammatory cells is immediately followed by the proliferation of HPCs during chronic liver injury,^[^ ^11d^ ^]^ and anti‐inflammatory agents can effectively reduce the activation of HPCs in liver injury models.^[^ ^22^ ^]^ The results of our previous work indicate that there is a correlation between the degree of inflammatory infiltration and the number of oval cells.^[^ ^4b^ ^]^ Previous research has reported that hepatic macrophage plays a key role in the HPC‐mediated regeneration of hepatocytes.^[^ ^8^ ^]^ Our work indicates that macrophages present potential interaction with HPCs through secreting high levels of TWEAK. TWEAK is a member of the TNF ligand superfamily and acts by binding to Fn14, the sole receptor of TWEAK, to initiate several intracellular signaling pathways, including NF‐*κ*B.^[^ ^23^ ^]^ Biologically, TWEAK has been shown to regulate numerous cellular processes including proliferation, differentiation, and cell survival and has also been described as a pro‐angiogenic and pro‐inflammatory factor.^[^ ^24^ ^]^ In the chronic liver disease model, TWEAK‐producing macrophages have been observed in close association with expanding ductal cells, demonstrating a primary role of macrophage‐generated TWEAK in initiating the activation of HPCs.^[^ ^25^ ^]^ The results from our and previous studies indicate that during the pathological process of liver injury, macrophages accumulate in the liver inflammatory environment and produce a high level of TWEAK, and create conditions that are favorable for the proliferation and aberrant differentiation of HPCs.

ID1 belongs to the HLH family of transcription factors, which bind to the bHLH transcription factors and inhibit DNA binding by these factors. ID1 has been proven to control the proliferation and differentiation of stem cells.^[^ ^14^ ^]^ It is also linked to tumorigenesis and is highly expressed in numerous types of cancers.^[^ ^26^ ^]^ It exerts its tumor‐promoting effects through different signaling pathways including the K‐Ras, BMP, PI3K/Akt, STAT3, MAPK, and TGF‐*β* pathways. Our research found that ID1 is highly expressed in malignant transformed HPCs induced by TWEAK secreted by macrophages. Clinical investigation also verified that ID1 was correlated to the malignant transformation of HPCs, and a high level of ID1 in HPCs suggested a poor prognosis in HCC patients. Further studies indicate that the high expression of ID1 promotes proliferation through regulating Rap1, PI3K‐AKT, MAPK, and TGF‐*β* signaling, and enhances the stemness of HPCs. We found that ID1 inhibits the transcription of HNF4*α* and Rap1GAP, which are the products of a bHLH target gene regulated by the E2A protein. HNF4*α* is a key mediator of HPC differentiation into hepatocytes. HNF4*α* deletion in hepatocytes is reported to cause hepatocyte differentiation defects and, in DEN‐treated mice, it induces accumulation of HPCs and formation of tumors showing HCC morphology.^[^ ^27^ ^]^ The HNF4*α*‐mediated hepatocyte differentiation program results in bipotential progenitors, creating a persistent pre‐neoplastic state primed for transformation by additional oncogenic mutation.^[^ ^28^ ^]^ Rap1GAP is a GTPase‐activating protein that inactivates Rap1‐GTP, which is the functional form of Rap1. Rap1GAP has been identified to be suppressed in cancers.^[^ ^16^, ^29^ ^]^ Our study indicates that Rap1GAP is inhibited by ID1, which leads to the upregulation of Rap1 signaling, finally promoting the proliferation of HPCs. Differentiation arrest and malignant proliferation ultimately lead to aberrant differentiation of HPCs into tumor‐initiating cells.

Taken together, we dynamically observe the transcription programs and signaling components in HPCs from the early stage until tumorigenesis in primary HCC by single‐cell sequencing technology. The results suggest that macrophage‐derived TWEAK promotes ID1 expression, which serves a key role in regulating the proliferation, differentiation, and malignant transformation of HPCs in hepatocarcinogenesis. ID1 suppresses differentiation and promotes cell proliferation in HPCs through the inhibition of HNF4*α* and Rap1GAP transcriptions. Finally, our findings indicate that the expression of ID1 in HPCs in clinical samples is also correlated with the recurrence of HCC patients. The results of our study provide a valuable resource, facilitate a deeper understanding of the mechanisms by which the hepatocarcinogenesis‐associated microenvironment regulates HPCs function, and identify a potential biomarker for the prognosis and therapy of HCC patients.

## 4 Experimental Section

### Animal Models and HCC Tissues

Male SD rats (8–10 weeks old, weighing 160–180 g) were obtained from Shanghai Laboratory Animal Center (Shanghai, China), and were housed in a pathogen‐free animal facility. Rats received DEN at 95 mg L^−1^ concentration through drinking water. At different time points, rats were sacrificed to obtain liver samples. To deplete macrophages, a 1 mL injection (≈20 mg) of clodronate‐encapsulated liposomes (Clodronate Liposomes, Amsterdam, The Netherlands) was administered beginning at week 8 of DEN treatment and continuing once weekly until euthanasia. The animal protocols were approved by the Naval Medical University Animal Care Committee.

Specimens of HCC tissues were obtained from 53 HCC patients who underwent hepatic resection at the Third Affiliated Hospital of Naval Medical University from 1997 to 2007. The clinical features were included in **Table** **1**. All the specimens were subjected to immunofluorescence analysis. Fresh hepatobiliary resected tumors were collected with informed consent from patients who were enrolled at the Third Affiliated Hospital of Naval Medical University. Prior informed consent was obtained, and the study protocol was approved by the Ethics Committee of the Third Affiliated Hospital of Naval Medical University.

**Table 1:** Demographic and baseline characteristics of 53 HCC patients

Factors	Value	Percent [%]
Age	49 ± 9.81	
Gender (male/female)	43/10	81.13/18.86
HbsAg (positive)	50/3	94.33/5.66
Cirrhosis	40/13	75.47/24.53
Maximum tumor diameter	5.40 ± 2.60	
Tumor number (multiple)	6	11.32
Tumor thrombosis	7	13.21
AFP [ng mL^−1^]	408.68 ± 455.74 (1.0–1000.0)	

### Preparation of Single‐Cell Suspensions

Liver and tumor tissues were processed immediately after being obtained from DEN‐treated rats and humans. Each sample was cut into small pieces (<1 mm), and the pieces were incubated with 1 mL of collagenase IV and 100 µL of DNase (Servicebio) for ≈15–30 min on a 37 °C shaker. Subsequently, 4 mL DMEM was added to dilute the suspension, and then a 70‐µm cell mesh was used to filter the suspension. After centrifugation at 250 g for 5 min, the supernatant was discarded, and the cells were washed twice with PBS. Then, the cell pellet was resuspended in 1 mL of ice‐cold red blood cell lysis buffer and incubated at 4 °C for 10 min. Next, 10 mL of ice‐cold PBS was added to the tube, which was then centrifuged at 250 g for 10 min. After decanting the supernatant, the pellet was resuspended in 5 mL of PBS containing 0.04% BSA. Finally, 10 µL of suspension was counted under a microscope. Trypan blue was used to quantify liver cells.

### Single‐Cell RNA Sequencing

Single‐cell RNA sequencing was performed by Shanghai NovelBio Co., Ltd. Chromium Single Cell 3’ Reagent v3 kits were used to prepare barcoded scRNA‐seq libraries according to the manufacturer's protocol. The cell suspension was loaded onto a chromium single‐cell controller instrument (10× Genomics) to generate single‐cell gel beads in the emulsion (GEMs). Approximately 12 000 cells were added to each channel, the target cell recovery was estimated to be 8000 cells. After the generation of GEMs, reverse transcription reactions were used to generate barcoded full‐length cDNA. The emulsions were disrupted using the recovery agent, and then cDNA clean‐up was performed with DynaBeads MyOne Silane Beads (Thermo Fisher Scientific). Next, cDNA was amplified by PCR for the appropriate number of cycles, which depended on the number of recovered cells. For single‐cell RNA‐Seq library preparation, single‐cell RNA‐seq libraries were constructed using Single Cell 30 Library Gel Bead Kit V2. Sequencing was performed on the Illumina HiSeq XTEN platform (Illumina, 150‐bp paired‐end protocol), according to the manufacturer's protocol.

### Analysis of scRNA Sequencing Data

For all analyses, the rat genome (ensemble v93) was used as a reference. For quality control, three quality measurements were calculated, including the number of total genes and transcripts and the percent of mitochondrion genes. Cells that expressed over 25% mitochondrion genes and 40 000 transcripts or lower than 500 genes were removed. The normalized and batch‐corrected data were imported into Seurat (v2.3.4) for downstream analysis and visualization. The dimensionality reduction was performed with principal component analysis (PCA). Unsupervised cell clusters of the same major cell type were selected for t‐distributed stochastic neighbor embedding (t‐SNE) analysis, graph‐based clustering, and marker analysis to identify the cell subtypes. The marker genes were calculated using the Seurat package FindMarkers function with the Wilcox rank‐sum test algorithm under the following criteria:1) logFC > 0.25; 2) *p* < 0.05; 3) min.pct > 0.1.

To identify DEGs between each group, the Seurat package FindMarkers function using the Wilcox rank sum test algorithm was used under the following criteria:1) logFC > 0.25; 2) *p* < 0.05; 3) min.pct > 0.1. Enrichment analysis for the functions of the DEGs was conducted based on KEGG pathways and GO analysis.

To identify cellular interactions, cell communication analysis was applied based on CellPhoneDB,^[^ ^30^ ^]^ a public database of ligands, receptors, and their interactions. Membrane, secreted, and peripheral proteins of the cluster were annotated. The mean and cell communication significance (*p* < 0.05) were calculated based on the interactions and the normalized cell matrix was obtained by Seurat normalization. The total number of ligand–receptor pairs between two clusters was obtained, and interactions were visualized as dot plots. Nichenet was utilized to deeper understand cell‐to‐cell interaction.^[^ ^12^ ^]^ This analysis included a large number of public databases (KEGG, ENCODE, PhoshoSite) to track the receptor's target in the provided dataset.

Single‐cell transcriptome datasets from liver tissues of healthy donors and patients with HCC were also collected. The liver tissue cells of healthy donors were from GSE136103,^[^ ^31^ ^]^ and the liver tissue cells of patients with HCC come from GSE149614.^[^ ^32^ ^]^

### Culture and Establishment of Rat Adult Liver and Human HCC Organoids

Primary HPCs were isolated from SD rats after treatment with DEN. Rats were anesthetized with pentobarbital sodium (30 mg kg^−1^). The liver was removed by surgical excision and then kept cold at 4 °C in basal medium in a 100‐mm dish. The liver was minced into pieces of roughly 0.5 mm^3^ using fine scissors and the tissue pieces were washed. 10 mL of digestion solution (0.1% type IV collagenase) prewarmed to 37 °C was added, and the digestion mixture was incubated on a shaker at 37 °C for ≈20–40 min. Then the supernatant was transferred to a fresh 50 mL centrifuge tube at 4 °C. The previous digestion steps were repeated for the remaining tissue. The supernatant was filtered through 70 and 40 µm mesh filters. The cells were then seeded into Cultrex reduced growth factor BME2 (basement membrane extract, Type 2; Pathclear) and suspended in advanced DMEM/F12 medium supplemented with 1:50 B‐27 (Gibco), 10 mM nicotinamide (Sigma), 1.25 mM N‐acetyl‐L‐cysteine (Sigma), recombinant rat EGF (50 ng mL^−1^, Peprotech, Rocky Hill, USA), recombinant human HGF (50 ng mL^−1^, Peprotech, Rocky Hill, USA), recombinant human FGF10 (100 ng mL^−1^, Peprotech, Rocky Hill, USA), recombinant human [Leu15]‐gastrin I (10 nM, Peprotech, Rocky Hill, USA), recombinant human RSPO1 (100 ng mL^−1^, MCE, NJ, USA), Y27632 (10 µM, Sigma‐Aldrich, SL, USA), and A8301 (1 mM, Tocris Bioscience, MN, USA). For in vitro differentiation induction, HPCs were cultured for 2 weeks with advanced DMEM/F12 medium supplemented with recombinant rat EGF (50 ng mL^−1^, Peprotech, Rocky Hill, USA), recombinant human FGF10 (100 ng mL^−1^, Peprotech, Rocky Hill, USA), recombinant human [Leu15]‐gastrin I (10 nM, Peprotech, Rocky Hill, USA), A8301 (50 nM, Tocris Bioscience, MN, USA), DAPT (10 µM, Sigma‐Aldrich, SL, USA), dexamethasone (3 µM, Sigma‐Aldrich, SL, USA).

Tumor tissue from HCC patients was minced and digested with 0.25% collagenase IV (Sigma) and 0.1 mg mL^−1^ DNase (Sigma) at 37 ℃. Tumor cells were then seeded into Cultrex reduced growth factor BME2 and added with advanced DMEM/F‐12 supplemented with 1:50 B‐27, 1:100 N‐2, 10 mM nicotinamide, 1.25 mM N‐acetyl‐L‐cysteine, 10 nM [Leu15]‐gastrin I, 10 µM forskolin, 5 µM A83‐01, 50 ng mL^−1^ EGF, 100 ng mL^−1^ FGF10, 25 ng mL^−1^ HGF, 100 ng mL^−1^ RSPO1, and 100 ng mL^−1^ Noggin (Peprotech). For drug treatment, sorafenib tosylate (Cat. No. S‐8502) was purchased from GLPBIO, dissolved in DMSO at 10 mM aliquots, and stored at −20 °C. Tumor organoids were plated at a density of 5 × 10^3^ cells in 15 µL BME2 droplets in order to form organoids. On day 6, sorafenib was added to the medium, and cell viability was measured after 6 days.

### In Vivo Tumorigenicity Experiments

Six‐week‐old male athymic BALB/c nu/nu mice were obtained from the Shanghai Experimental Animal Center, Chinese Academy of Sciences. Mice were maintained under pathogen‐free conditions and treated in accordance with the institutional animal welfare guidelines of the Naval Medical University. For the assay to assess tumorigenicity, HPC‐derived organoids from different time points of DEN‐treated rats’ livers were cultured within 2 weeks and 200 organoids were injected subcutaneously into the right axilla of the mice. At the end of 2 months, the mice were sacrificed for analysis.

### Immunohistochemical Staining and Immunofluorescence

The slides were deparaffinized in xylene and rehydrated through gradient alcohol. Endogenous peroxidase was then inactivated with 3% hydrogen peroxide at room temperature for 20 min (only for IHC). Next, the antigen retrieval was enhanced by autoclaving the slides in 0.1 mol L^−1^ citrate buffer (pH 6.0) for 2 min. After washing with PBS, the sections were blocked with 3% BSA at 37 °C for 30 min. The slides were then incubated overnight at 4 °Cwith primary antibodies. Subsequently, the HRP‐conjugated goat antibody and DAB (Dako, Carpinteria, CA, USA) or fluorescent‐labeled secondary antibodies were used. Images were captured with the microscope. At least three random areas per slide were selected to count the number of positively stained cells. IHC analysis was performed using the following antibodies: EpCAM (diluted 1:100, Abcam, Cambridge, UK), Sox9 (diluted 1:200, Abcam, Cambridge, UK), AFP (diluted 1:200, Abcam, Cambridge, UK), ID1 (diluted 1:200, Santa Cruz Biotechnology, Texas, USA), CD68 (diluted 1:200, Abcam, Cambridge, UK), and PCNA (diluted 1:200, Abcam, Cambridge, UK). For immunofluorescence, sections were incubated with primary antibodies against EpCAM (diluted 1:100, Abcam, Cambridge, UK), Sox9 (diluted 1:200, Abcam, Cambridge, UK), CD24 (diluted 1:100, Proteintech Group, Rosemont, IL, USA), CLDN4 (diluted 1:100, Proteintech Group, Rosemont, IL, USA). ALB (diluted 1:200, Abcam, Cambridge, UK), HNF‐4*α* (diluted 1:100, Abcam, Cambridge, UK), AFP (diluted 1:200, Abcam, Cambridge, UK), ID1 (diluted 1:200, Santa Cruz Biotechnology, Texas, USA), *α*‐SMA (diluted 1:200, Abcam, Cambridge, UK), VWF (diluted 1:200, Abcam, Cambridge, UK), CD68 (diluted 1:200, Abcam, Cambridge, UK), TWEAK (diluted 1:200, Affinity Biosciences, Jingsu, China), Fn14 (diluted 1: 200, Santa Cruz Biotechnology, Texas, USA), cleaved‐caspase3 (diluted 1: 200, Cell Signaling Technology, Beverly, MA, USA), and PCNA (diluted 1:200, Abcam, Cambridge, UK).

### qRT‐PCR

Total RNA was extracted using a HiPure Total RNA Plus Micro Kit (Magen, China) and reverse transcribed into cDNAs using Bestar qPCR RT Kit with a total reaction volume of 20 µL. qPCR was conducted using the Bestar one‐step RT qPCR kit (Sybr Green) (DBI, China) according to the manufacturer's instructions. The running parameters for qPCR were set as follows: 95 °C for 1 min (pre‐denaturation), 95 °C for 15 s (denaturation), 60 °C for 30 s (annealing), and 72 °C for 15 s (extension) for 40 cycles. GAPDH was used as an internal reference.

### Bulk RNA Sequence Analysis

Total RNA was extracted from each tissue sample using TRIzol (Life Technologies, Grand Island, NY, USA), according to the protocol provided by the manufacturer. Five micrograms of RNA of each sample were individually used for the construction of transcriptome libraries, using IlluminaTruSeq RNA Sample Preparation Kit (Illumina, San Diego, CA, USA), and sequenced using IlluminaHiSeq 2000, according to the manufacturer's instructions. Q20 was used as a quality control standard to filter raw reads. After filtering the low‐quality reads, the adaptors of high‐quality reads were removed, and then clean reads were aligned to the rat genome, using the UCSC rat reference [build Rn4]. The fragments per kilobase of exon model per million mapped reads (FPKM) values were calculated according to the counts and lengths of genes. The differentially expressed genes with the fold change (FC) ≤ 0.5 or FC ≥ 2 and *p* < 0.05 were selected. For gene GSEA analysis, normalized values of RNA‐seq data (FPKM) were rank‐ordered by fold change as input. The analysis was performed using GSEA (version: 4.2, https://www.gsea‐msigdb.org/gsea/index.jsp) software. The sequencing was performed by Biomarker (Beijing, China).

Transcriptome datasets were also collected from TCGA‐LIHC. The transcriptome of 52 HCC adjacent non‐tumor tissues was from GSE76427.^[^ ^33^ ^]^

### Quantitative Proteomics

The protein was extracted from HPC‐derived organoids. Label‐free quantitative proteomics analysis was performed by Jingjie PTM BioLab Co Inc. (Hangzhou, China). Systematic bioinformatics analysis was then performed on all identified proteins. The analysis mainly included quantification of protein expression and differential expression analysis. Then, based on the differentially expressed proteins, protein functions were classified by GO enrichment analysis and KEGG enrichment analysis.

### Macrophages Isolation and Culture

Rats were anesthetized with pentobarbital sodium (30 mg kg^−1^). The liver was removed and perfused in situ via the portal vein with warmed (37 °C) Hanks’ balanced salt solution (HBSS), followed by 0.1% collagenase IV. Then livers were minced into pieces and treated with digestion solution (0.1% type IV collagenase) at 37 °C for 30 min. Then cells were filtered through 70 µm mesh filters. Nonparenchymal cells were separated from hepatocytes by three 2‐min centrifugations at 50 g. Nonparenchymal cells were suspended in HBSS and layered onto a 60/30% two‐step Percoll gradient (Sigma) and centrifuged at 1600 g at 4 °C for 15 min. Macrophages in the middle layer were collected and allowed to attach to cell culture plates in DMEM with 10% FBS, 100 U mL^−1^ penicillin, and 100 µg mL^−1^ streptomycin at 37 °C for 1 h. Nonadherent cells were removed by replacing the culture medium. Cells and conditioned medium (CM) were collected for further experiments.

### Enzyme‐Linked Immunosorbent Assay

Conditioned medium (CM) was collected from macrophages. The levels of TWEAK in CM were determined using an enzyme‐linked immunosorbent assay (ELISA) kit (Codino (Wuhan) Biotechnology Co., Ltd, China), according to the manufacturer's instructions.

### siRNA and Lentiviral Transfection

For Tnfsf12 interference, three siRNA candidates were produced by Hanbio (Shanghai, China). 1 × 10^5^ cells per well were grown in 6‐well plates and transfected with siRNAs by using LipoFiter transfection reagent (Hanbio, Shanghai, China) for 36 h. For Tnfrsf12a, Id1, and Tcf3 interference, three shRNA candidates with Tnfrsf12a, Id1, and Tcf3 target sequences were designed by Hanbio (Shanghai, China). Scramble shRNA served as the negative control. pGCL‐GFP lentiviral particles encoded GFP and shRNAs (Obio Technology, Shanghai, China). For Id1 overexpression, the Id1 cDNA cloned by PCR was inserted into CMV‐MCS‐IRES‐EGFP lentiviral vectors (Hanbio, Shanghai, China). The recombinant lentivirus with Id1 coding sequence was produced by co‐transfection of 293T cells with plasmids PSPAX2 and PMD2G using LipoFiterTM (Hanbio, Shanghai, China). After lentiviral infection, the expressions of Fn14, ID1, and E2A were analyzed by western blotting. The sequences of Tnfsf12 siRNA and the scramble control are shown in Table S4, Supporting Information.

### Western Blotting

Protein samples were collected and electrophoresed on a 10% sodium dodecyl sulfate/polyacrylamide gel in 1× tris‐glycine buffer. Proteins were then transferred to nitrocellulose membranes and incubated with primary antibodies overnight at 4 °C. Thereafter, the nitrocellulose membranes were incubated with secondary antibodies for 1 h at RT. The immunoreactive proteins were detected by enhanced chemiluminescence substrate and the blot was scanned. The primary antibodies used were ID1 (diluted 1:1000, Santa Cruz Biotechnology, Inc, Texas, USA), FN14 (diluted 1:1000, Santa Cruz Biotechnology, Inc, Texas, USA), TWEAK (diluted 1:1000, Affinity Biosciences, Jingsu, China), I A*α* (diluted 1:1000, Cell Signaling Technology, Beverly, MA, USA), p‐I*κ*B*α* (diluted 1:1000, Cell Signaling Technology, Beverly, MA, USA), p65 (diluted 1:1000, Cell Signaling Technology, Beverly, MA, USA), p‐p65 (diluted 1:1000, Cell Signaling Technology, Beverly, MA, USA), Rap1 (diluted 1:200, Santa Cruz Biotechnology, Texas, USA), AKT (diluted 1:1000, Proteintech Group, Rosemont, IL, USA), p‐AKT (diluted 1:1000, Proteintech Group, Rosemont, IL, USA), p38 MAPK (diluted 1:1000, Cell Signaling Technology, Beverly, MA, USA), p‐p38 MAPK (diluted 1:1000, Cell Signaling Technology, Beverly, MA, USA), STAT3 (diluted 1:500, SAB, Maryland, USA), p‐STAT3 (diluted 1:500, SAB, Maryland, USA), Sox9 (diluted 1: 1000, Abcam, Cambridge, UK), EpCam (diluted 1: 1000, Abcam, Cambridge, UK), CD133 (diluted 1:1000, Cell Signaling Technology, Beverly, MA, USA), ALDH1A1 (diluted 1:500, Abcam, Cambridge, UK), CD44 (diluted 1:2000, Abcam, Cambridge, UK), PCNA (diluted 1:2000, Proteintech Group, Rosemont, IL, USA), HNF4*α* (diluted 1: 1000, Abcam, Cambridge, UK), E2A (diluted 1: 200, Santa Cruz Biotechnology, Texas, USA), Rap1GAP (diluted 1: 1000, Abcam, Cambridge, UK), and GAPDH (diluted 1:5000, Bioworld Technology, MN, USA).

### TUNEL Staining

The samples were immersed and washed in xylene twice for dewaxing, then hydrated in a gradient of alcohol (100–70%, 3 min each step). Next, the samples were treated with proteinase K for 30 min at 37 °C and washed in PBS twice. Slides were wiped dry and 50 µL TUNEL reaction mixture was added to the sample. After sealing with sealing film, samples were incubated in a dark wet box for 1 h at 37 °C followed by washing in PBS three times. Slides were wiped dry and 50 µL Converter‐POD was added to the samples. After applying the sealing film, samples were placed in a dark wet box again for 1 h at 37 °C, then washed three times in PBS. Next, samples were treated with 50 µL DAB substrate in a dark wet box for 1 h at 37 °C and washed in PBS three times. Subsequently, sections were dehydrated in an alcohol gradient (80–100%), cleared in xylene, and sealed with neutral balsam. Apoptosis of cells was then examined under an electron microscope.

### Immunoprecipitation

Primary HPCs were lysed in IP lysis/wash buffer supplemented with phosphatase and protease inhibitors. The total protein concentration of the supernatants was quantified using a BCA protein assay kit (Beyotime, Shanghai, China) after centrifugation for 10 min at 13 000 g at 4 °C. One milligram of total protein was mixed with 2 µg of the indicated primary antibody or isotype control IgG, and the mixture was shaken on a rotating shaker at 4 °C overnight. Immunoprecipitates were collected and washed three times with lysis buffer, and proteins were analyzed by western blot.

### Cleavage Under Targets and Tagmentation (CUT&Tag)

To confirm that E2A binds to the promoter of HNF4*α*, the CUT&Tag assay was performed using a NovoNGS CUT&Tag 3.0 High‐Sensitivity Kit (Novoprotein, #N259‐YH01) following the manufacturer's instructions. Briefly, cells were harvested, counted, and centrifuged for 5 min at 600× g at RT. Next, cells were washed in 300 µL wash buffer and collected by centrifugation at 600× g for 5 min. Then cells were resuspended in 90 µL wash buffer and 10 µL ConA beads, and incubated on a magnetic stand for 10 min at RT. Then, primary antibody incubation was performed on a rotating platform for 2 h at RT. A corresponding secondary antibody was incubated with the cells for 30 min at RT. Then, A 1:100 dilution of pA‐Tn5 adapter complex was prepared in ChiTag buffer on a rotating platform for 1 h at RT Buffer. The cells were washed three times for 5 min in ChiTag buffer to remove unbound pA‐Tn5 protein. Next, the cells were resuspended in 50 µL tagmentation buffer and incubated at 37 °C for 1 h. To stop tagmentation, 1 µL of 10% SDS was added and then incubated at 55 °C for 10 min. DNA fragments were extracted by Tagment DNA extract beads. The beads were washed with 80% ethanol and mixed well with a pipette tip. The supernatant was removed. After drying, 37 µL TE buffer was added to each sample to dissolve the DNA. The final products were subjected to qPCR analysis. The primer sequences for CUT&Tag detection are provided in Table S5, Supporting Information.

### Implementation of Single‐Sample Gene Set Enrichment Analysis (ssGSEA)

In brief, the cell types composition of liver tumor microenvironment was quantified by ssGSEA in R package gsva. The gene signatures expressed by different cell populations (B cells, T cells, macrophages, dendric cells, HPCs, myofibroblasts, neutrophils, and NK cells) are included in Table S6, Supporting Information. The scores were based on analysis of transcriptomic markers – that is, transcriptomic features that were strongly, specifically, and stably expressed in a unique cell population.^[^ ^17^, ^34^ ^]^

### Statistical Analysis

Statistical analysis was performed with Prism software (GraphPad Prism 8.0 software). Student's *t*‐test was used to compare the mean values between the two groups. Multiple group comparisons were performed using the one‐way ANOVA method. The data were expressed as mean ± SD. A difference of at least *p* < 0.05 was considered statistically significant. *p < 0.05; **p < 0.01; ***p < 0.001, and ns: not significant.

### Ethical Approval and Consent

The present study was approved by the Ethics Committee of the Third Affiliated Hospital of Naval Medical University. The animal protocols were approved by the Naval Medical University Animal Care Committee.

## Conflict of Interest

The authors declare no conflict of interest.

## Author Contributions

L.W.T. and G.L. conceived and designed experiments. L.W.T. performed the analysis of scRNA, bulk RNA, and proteomic sequencing data. L.W.T. and H.X.J. analyzed data and wrote the manuscript. L.W.T., G.L., F.S.Y., Y.H.X., P.H.Y., Y.X., Z.S.C., J.J.H., Y.F., and Z.Q.D. performed the in vitro and in vivo studies. W.L.X. and H.Z.P. supervised the project. All authors read and approved the final manuscript.

## Supporting information

Supporting Information

Supplemental Table 1

Supplemental Table 2

Supplemental Table 3

Supplemental Table 6

## Data Availability

The data that support the findings of this study are available from the corresponding author upon reasonable request.
