# Supplementary material for: TWEAK Signaling‐Induced ID1 Expression Drives Malignant Transformation of Hepatic Progenitor Cells During Hepatocarcinogenesis
Source: Adv Sci (Weinh). 2023 Apr 21;10(18):2300350. doi: 10.1002/advs.202300350 (PMC10288241; doi:10.1002/advs.202300350)
Supplement: Supplementary file 1 — Supporting Information [file ADVS-10-2300350-s005.pdf]

ADVANCED SCIENCE
Open Access

Supporting Information

for *Adv. Sci.*, DOI 10.1002/advs.202300350

TWEAK Signaling-Induced ID1 Expression Drives Malignant Transformation of Hepatic Progenitor Cells During Hepatocarcinogenesis

Wenting Liu, Lu Gao, Xiaojuan Hou, Shiyao Feng, Haixin Yan, Hongyu Pan, Shichao Zhang, Xue Yang, Jinghua Jiang, Fei Ye, Qiudong Zhao, Lixin Wei and Zhipeng Han**

Supplemental Information

TWEAK signaling-induced ID1 expression drives malignant transformation of hepatic progenitor cells during hepatocarcinogenesis

Wenting Liu[#1,2], Lu Gao[#1,2], Xiaojuan Hou[#1,2], Shiyao Feng[3], Haixin Yan[3], Hongyu Pan[4], Shichao Zhang[4], Xue Yang[1,2], Jinghua Jiang[1,2], Fei Ye[1,2], Qiudong Zhao[1,2], Lixin Wei [*1,2], Zhipeng Han [*1,2]

[1]Tumor Immunology and Gene Therapy Center, Third Affiliated Hospital of Naval Medical University, Shanghai, 200438, China

[2]Key Laboratory on Signaling Regulation and Targeting Therapy of Liver Cancer, Ministry of Education, Eastern Hepatobiliary Surgery Hospital/National Center for Liver Cancer, Naval Medical University, Shanghai, 200438, China

[3]Department of Urology, Second Affiliated Hospital, Anhui Medical University, Hefei, 230601, China.

[4]Department of Hepatic Surgery, Third Affiliated Hospital of Naval Medical University, Shanghai, 200438 China

*Correspondence to: Zhipeng Han and Lixin Wei, Tumor Immunology and Gene Therapy Center, Third Affiliated Hospital of Naval Medical University, 225 Changhai Road, Shanghai, 200438 China. E-mail: hanzhipeng0311@126.com and weilixin_smmu@163.com

[#] These authors contributed equally to this work.

Supplemental results

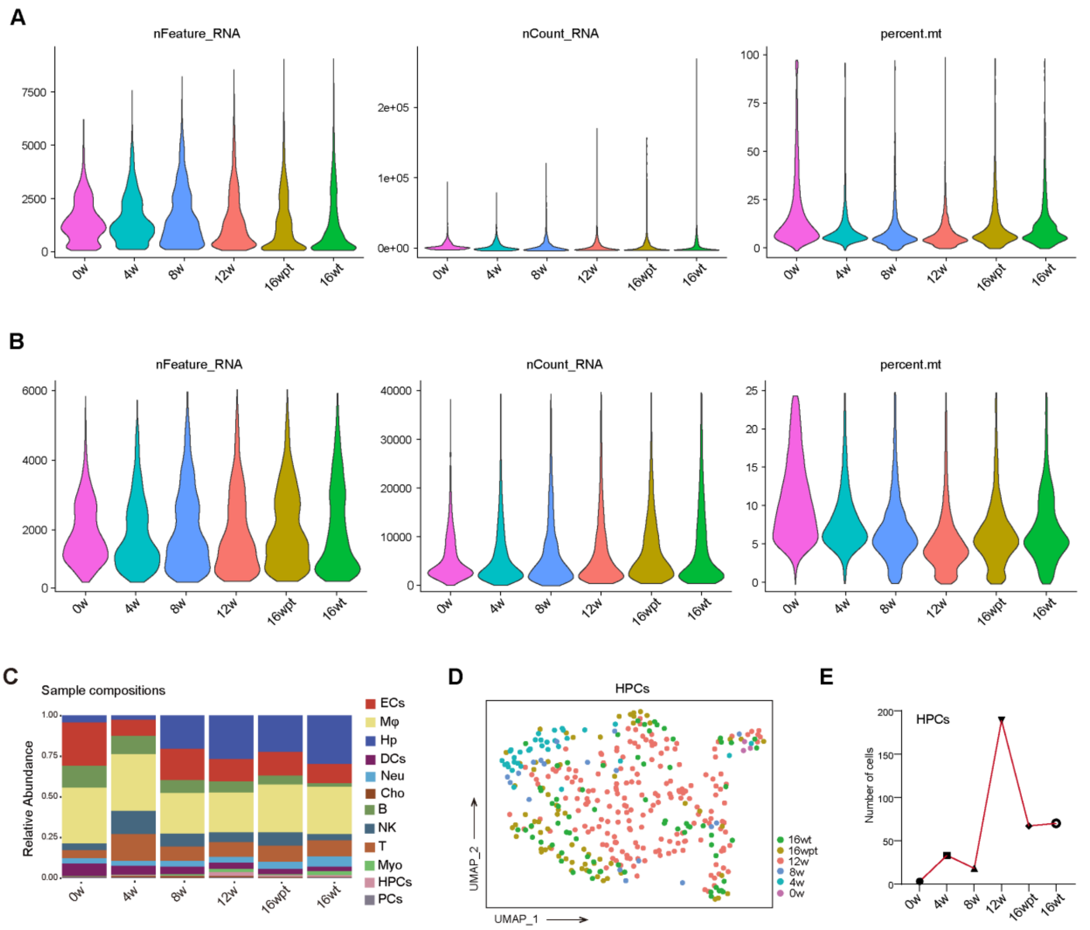

Figure S1

A. Violin plot (Vlnplot) showing nFeature_RNA, nCount_RNA and percent.mt in scRNA sequence data before quality control. B. Vlnplot showing nFeature_RNA, nCount_RNA and percent.mt in scRNA sequence data after quality control (nFeature_RNA ≦ 6000, nCount_RNA ≦ 40000, percent.mt ≦ 25). C. Percentage distribution of cell types in the six DEN-induced rat liver samples. D. UMAP showing the HPCs at different time points of DEN treatment. E. The number of HPCs was observed in rat liver at different time points of DEN induction.

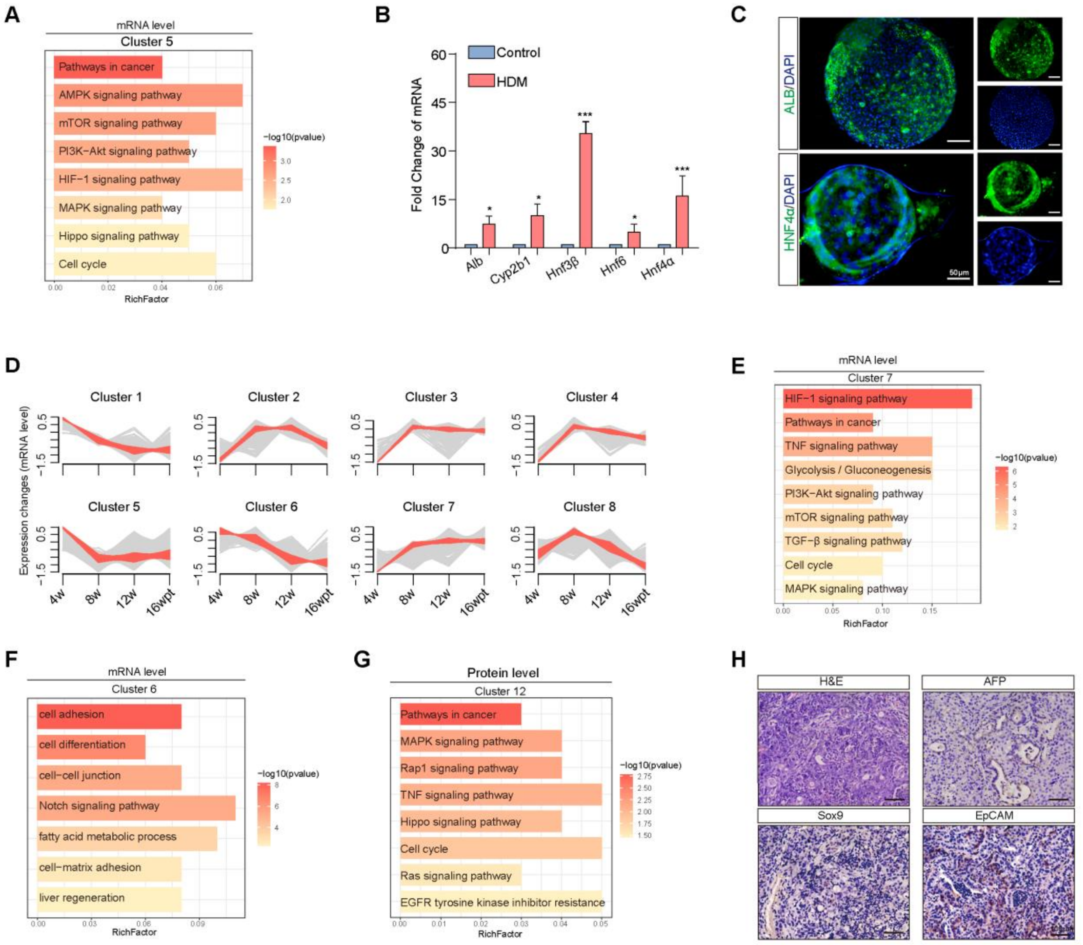

Figure S2

A. Bar graph showing the enriched pathways identified by KEGG analysis of the genes in cluster 5 by scRNA sequencing data. B and C. The *in vitro* differentiation induction system was performed in HPC-derived organoids, then RT-PCR was used to detect the mRNA levels of hepatocyte marker genes. Data are presented as mean±SD. *p<0.05, ***p<0.001. The protein levels of ALB and HNF4α were examined by immunofluorescence analysis. Nuclei were stained with DAPI (blue). D. Fuzzy C-means clustering identified 8 distinct temporal patterns of gene expression. E and F. Bar graph showing the enriched pathways identified by KEGG analysis of the genes in clusters 7 and 6. G. Bar graph showing the enriched pathways identified by KEGG analysis of the protein in cluster 12. H. Images of H&E-stained liver sections showing the histological structure of the subcutaneous tumor from 16wpt organoids. EpCam, Sox9 and AFP expression was detected in the tumor from 16wpt organoids by IHC analysis.

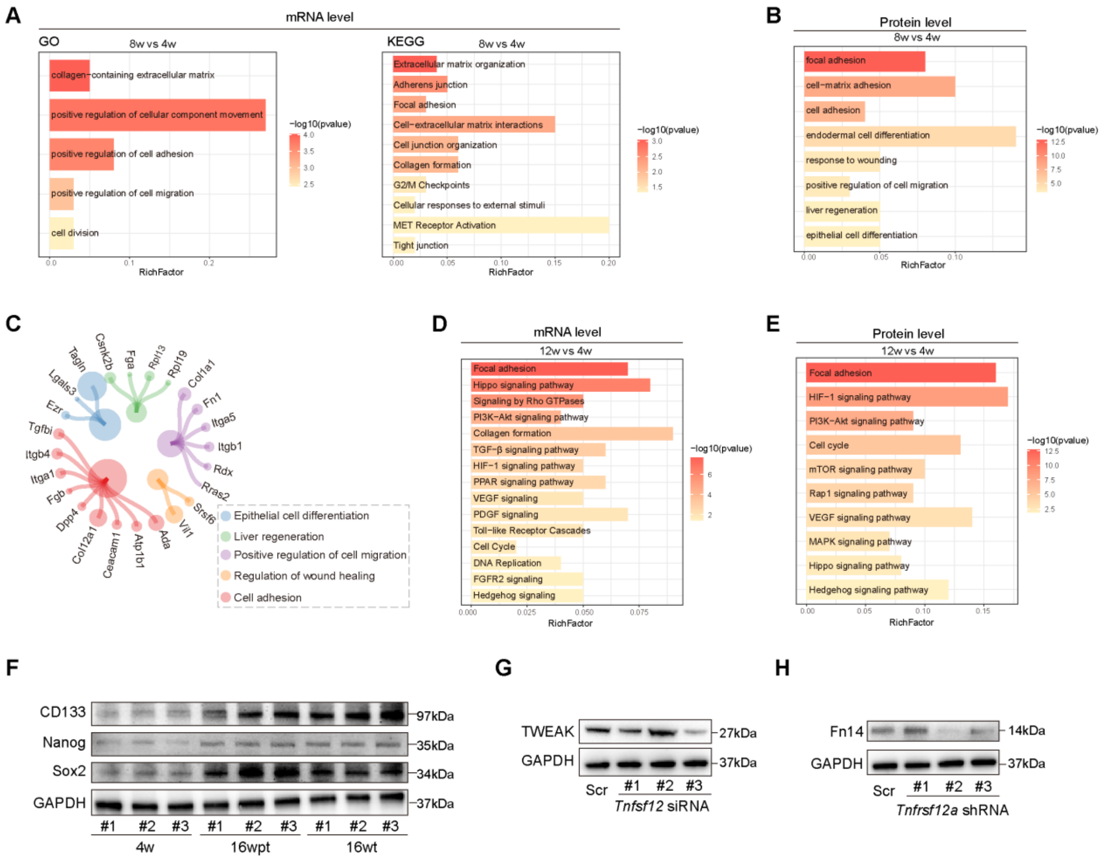

Figure S3

A. Bar graph showing the enriched pathways identified by GO and KEGG analysis of scRNA sequencing data from HPCs of rats at 8 weeks of DEN treatment. B and C. Bar graph showing the enriched terms identified by GO analysis of proteomic data from HPCs of rats at 8 weeks of DEN treatment. Representative proteins were indicated in chord graph. D. Bar graph showing the enriched pathways identified by KEGG analysis of scRNA sequencing data from HPCs of rats at 12 weeks of DEN treatment. E. Dot plot showing KEGG enrichment analysis of proteomic data from HPCs of rats at 12 weeks of DEN treatment. F. CSC markers (SOX2, Nanog and CD133) expression in HPCs of rats and 4w, 16wpt and 16wt was detected by western blotting. GAPDH was used as internal reference. G and H. The inhibitory effect of siRNA or shRNA on TWEAK and Fn14 expression was evaluated by western blotting. GAPDH was used as internal reference.

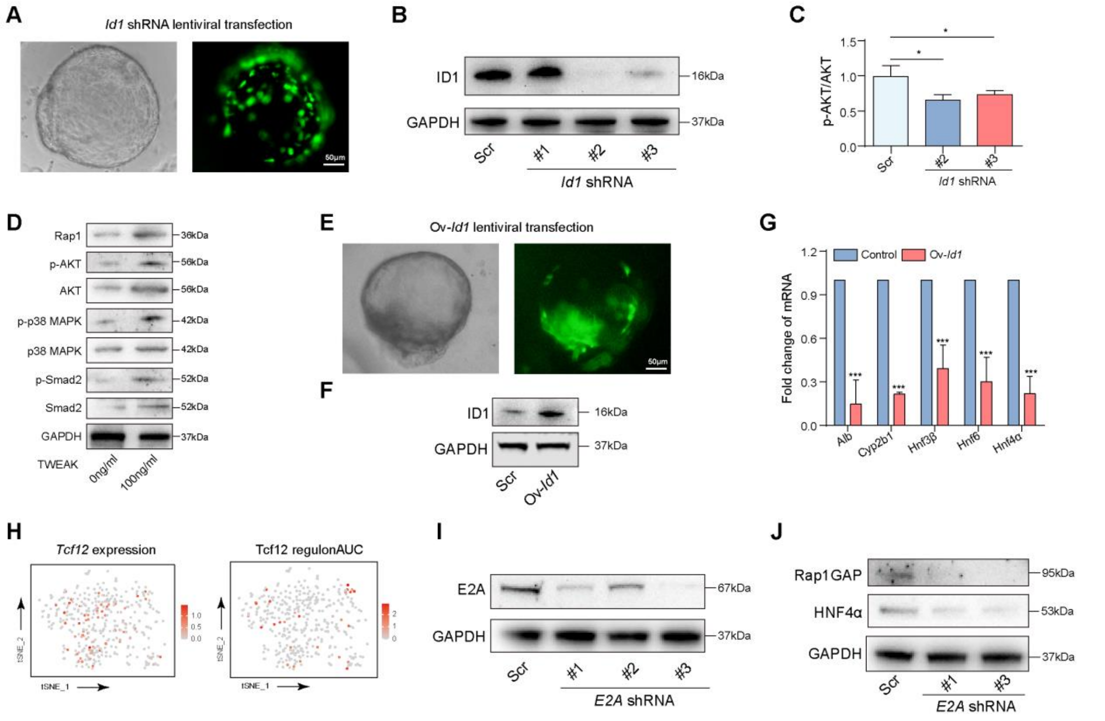

Figure S4

A. Transfection of *Id1* shRNA lentivirus (GFP-labeled) was detected by immunofluorescence analysis in organoids derived from rat adjacent non-tumor liver HPCs at 16 weeks of DEN treatment. B. The inhibitory effect of shRNA on ID1 expression was evaluated by western blotting. GAPDH was used as internal reference. C. The ratio of p-AKT and AKT expression was evaluated in each group. Data are presented as mean±SD. *p<0.05. D. HPCs were treated with TWEAK (100ng/ml) and then western blot was employed to detect the expression of Rap1, PI3K-AKT, MAPK, Smad2 and their phosphorylation level. E. Transfection of a lentivirus (GFP-labeled) overexpressing *Id1* (Ov-*Id1*) was detected by immunofluorescence analysis in organoids derived from rat liver HPCs at 8 weeks of DEN treatment. F. ID1 expression was evaluated by western blotting. GAPDH was used as internal reference. G. The *in vitro* differentiation induction system was performed in HPC-derived organoids in each group. RT-PCR was used to detect the mRNA levels of hepatocyte marker genes. Data are presented as mean±SD. ***p<0.001. H. t-SNE plot showing the expression and regulonacvtity of E2A family member TCF12 in HPCs from liver of rat HCC model by scRNA sequencing. I. The inhibitory effect of shRNA on E2A

expression was evaluated by western blotting. GAPDH was used as internal reference. J. HNF-4α and Rap1GAP expression was evaluated by western blotting. GAPDH was used as internal reference.

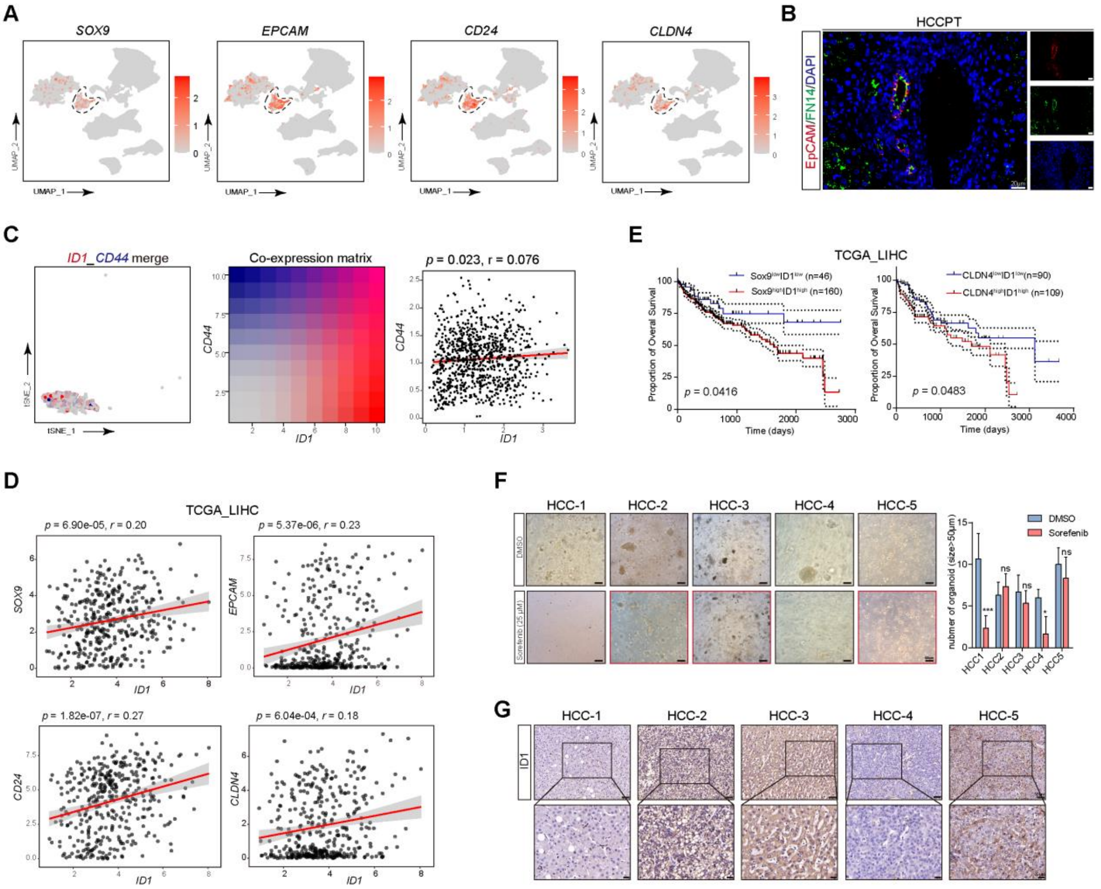

Figure S5

A. UMAP plot showing the subgroup of HPCs expressing the marker genes SOX9, EPCAM, CD24 and CLDN4. B. Fn14 expression (green) in HPCs (Epcam, red) from liver of HCCPT was detected by immunofluorescence analysis. Nuclei were stained with DAPI (blue). C. tSNE plots showing the co-expression of CD44 and ID1 in HPCs. Red/bule dots represent ID1+ only/CD44+ only, respectively, and pink dots indicate HPCs co-expressing ID1 and CD44; middle panel showing the co-expression matrix across HPCs with different expression of ID1 and CD44; right panels showing the correlation (Pearson) between ID1 and CD44 in HPCs. D. Correlation of ID1

expression with HPCs markers (SOX9, EpCAM, CD24 and CLDN4) (TCGA LIHC data). Each dot represents a patient (Pearson's correlation analysis). E. Survival rate was analyzed in each group of HCC patients (Kaplan-Meier method), red is high expression, and blue is low proportion. $Sox9^{high}ID1^{high}$ (Sox9≧1.8035 and ID1≧3.0517), $Sox9^{low}ID1^{low}$ (Sox9<1.8035 and ID1<3.0517), $CLDN4^{high}ID1^{high}$ (CLDN4≧1.2442 and ID1≧3.0517) and $CLDN4^{low}ID1^{low}$ (CLDN4<1.2442 and ID1<3.0517). The p-value was obtained by the log-rank multiple comparisons test. F. Tumor organoids were obtained from 5 HCC patients and cell viability was observed in control and sorafenib (25μM)-treated group. Number of organoids was calculated, data are presented as mean±SD, *p<0.05, ***p<0.001, ns, non-significant. G. ID1 expression in liver tumor tissues was detected by IHC.

Table S1 The genes in each cluster by scRNA sequence data were shown in table

Table S2 The genes in each cluster by bulk RNA-seq analysis were shown in table

Table S3 The proteins in each cluster by proteomic analysis were shown in table

Table S4 Sequences for *Tnfsf12* siRNA

	Forward	Reverse
NC	UUCUCCGAACGUGUCACGUTT	ACGUGACACGUUCGGAGAATT
siRNA1	CUCGAAGAAGUGUUUCUAATT	UUAGAAACACUUCUUCGAGTT
siRNA2	GGUGUGGAUGGGACAGUGATT	UCACUGUCCCAUCCACACCTT
siRNA3	GGAAGGCAGUCUACCUGAATT	ACGUGACACGUUCGGAGAATT

Table S5 Primers for CUT&Tag qPCR analysis

Primers	Forward (5'-3')	Reverse (5'-3')
Primer A	CTCTGCTGCTGTGTGTGGG	TGTCGTCCTGTAGGCTCCAT
Primer B	AGAAAGGAAGGGTCCCCGTA	CTACCCAGGGCAAGATCACG
Primer C	GGATTCTTTGCTTTGACTCACCA	TTAACTGTGCTTCCGTAATCTCT

Primer D	GTCTCAGTTTGCATCCCGGT	CCTGCTAACCTCAGGAACGG

Table S6 The gene signatures expressed by different cell populations (B cells, T cells, Macrophage, Dendric cells, HPCs, Myofibroblasts, Neutrophils, NK cells) were shown in table
